# Atom-Atom-Path similarity and Sphere Exclusion clustering: tools for prioritizing fragment hits

**DOI:** 10.1186/s13321-015-0056-8

**Published:** 2015-03-25

**Authors:** Alberto Gobbi, Anthony M Giannetti, Huifen Chen, Man-Ling Lee

**Affiliations:** Small Molecule Discovery, Discovery Chemistry, Genentech, 1 DNA Way, 94080 South San Francisco, CA USA; Small Molecule Discovery, Biochemical and Cellular Pharmacology, Genentech, 1 DNA Way, 94080 South San Francisco, CA USA

**Keywords:** Command line program, Clustering, Fragment screening, Hit prioritization, Similarity, Sphere exclusion

## Abstract

**Background:**

After performing a fragment based screen the resulting hits need to be prioritized for follow-up structure elucidation and chemistry. This paper describes a new similarity metric, Atom-Atom-Path (AAP) similarity that is used in conjunction with the Directed Sphere Exclusion (DISE) clustering method to effectively organize and prioritize the fragment hits. The AAP similarity rewards common substructures and recognizes minimal structure differences. The DISE method is order-dependent and can be used to enrich fragments with properties of interest in the first clusters.

**Results:**

The merit of the software is demonstrated by its application to the MAP4K4 fragment screening hits using ligand efficiency (LE) as quality measure. The first clusters contain the hits with the highest LE. The clustering results can be easily visualized in a LE-over-clusters scatterplot with points colored by the members’ similarity to the corresponding cluster seed. The scatterplot enables the extraction of preliminary SAR.

**Conclusions:**

The detailed structure differentiation of the AAP similarity metric is ideal for fragment-sized molecules. The order-dependent nature of the DISE clustering method results in clusters ordered by a property of interest to the teams. The combination of both allows for efficient prioritization of fragment hit for follow-ups.

**Graphical abstract:**
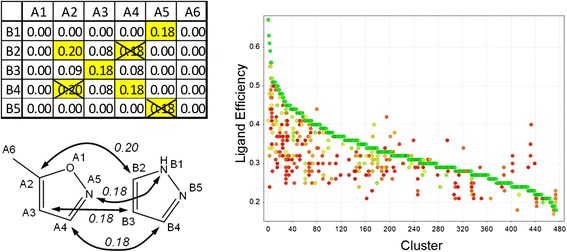
AAP similarity computation and DISE clustering visualization.

**Electronic supplementary material:**

The online version of this article (doi:10.1186/s13321-015-0056-8) contains supplementary material, which is available to authorized users.

## Background

Screening of low-affinity and low-molecular weight fragments has become a powerful approach to identify lead matter and to initiate medicinal chemistry programs [1]. It has been shown that binding affinity does not increase linear with the molecular size [2]. Fragments are more likely to bind with high ligand efficiencies [3,4] and can reveal important interactions required in more mature drug-like molecules. Choosing fragment hits for structure determination and chemical follow-up involves the evaluation of many project-specific parameters. Ligand efficiency (LE) is often used as one measure for assessing the quality of initial fragment hits.

Due to the generally higher hit rates of fragment screens (1-10%) most campaigns on libraries of 1,000-5,000 compounds will produce more hits, than downstream structural biology and chemistry resources can absorb for hit-to-lead development. To increase the chance of success, careful prioritization of the initial hits by a group of experienced specialists from different areas of drug discovery is important for advancing the most promising fragment hits. Fragment hit triage in advance of a structure determination typically weighs the LE parameter. However, many other properties including affinity, selectivity, and most importantly the chemical structure of the compound need critical consideration. In addition, fragment libraries often contain related molecules providing initial SAR and confidence in scaffold types. Clustering hit sets helps bring related molecules and features together for consideration, but the cluster order is usually determined by the algorithm and is independent of other factors such as LE. This results in a functional randomization of the order of the experimental data making trends harder to identify. To direct the attention of the specialists to the most promising hits we have employed a directed clustering method using a new similarity algorithm, to group hits with respect to both structure and data.

The Directed Sphere Exclusion (DISE) algorithm [5] used for the fragment clustering is an extension of the Sphere Exclusion (SE) algorithm [6,7]. The first step consists of ordering input molecules by a non-structural parameter such as LE, which is followed by a diverse subset selection using the SE algorithm. The selected molecules are considered as cluster seeds. In the final step, the remaining molecules are assigned to the cluster seeds. The investigator has the choice of two assigning rules: Assign the remaining molecules to (a) the first cluster seed whose similarity to the candidate molecule is within the specified threshold or (b) the most similar cluster seed.

Sorting the input molecules according to decreasing LE ensures that fragments having the highest LE are evaluated first in the cluster seed selection. Assigning the remaining molecules to the cluster seeds using rule (a) yields clusters with decreasing maximum LE while using rule (b) produces clusters that are structurally more consistent. In either case, the DISE clustering places the fragment clusters with favorable LE on top of the list, thus, directing the focus of the review team to the most promising clusters. Consequently, molecules with low LE (LE < 0.3, typically 30-50% of hits in most screens) are either moved towards the bottom of the list or included in clusters with a higher LE compound as cluster seeds, and therefore do not distract the team from clusters that are of higher interest. To our knowledge there are no other clustering methods that allow the clustering to be influenced by a target property. Frequently used clustering methods in chemical informatics [8] include hierarchical clustering, Jarvis Patrick clustering and k-means clustering. They are either order independent or aim to minimize the order dependency (k-means clustering). This means that their results depend only on the similarity matrix and cannot be influenced by external target properties.

Equally important is the choice of similarity metric. Many similarity metrics have been published in the past [9-15]. For fragment hit clustering, the similarity metric should meet the following requirements: (1) To satisfy the perception of the medicinal chemists, the similarity metric should put a high weight on common substructures. (2) The similarity metric should be sensitive to small changes in substitution. The latter is important because fragments are small in comparison to High Throughput Screening (HTS) compounds. Therefore, small changes have a strong effect on similarity in fragments.

Fingerprint based similarity methods generate fingerprints by systematically recording patterns present in compounds. Generally linear or circular patterns originating from each atom are used. The similarity of two compounds is then computed by comparing the patterns present or absent in each [9-17]. Their relative orientation is not stored while recording the patterns. Consequently, the relative orientation of the patterns in the compounds is not taken into consideration when computing the similarity. When comparing compound **1a** using linear fingerprints to **1b** a low similarity is found due to the difference of the central atom on the piperidine/piperazine ring as noted by Stahl *et al.* [15]. When using circular fingerprints compounds **1a** and **1b** are nearest neighbors. However, because circular fingerprints have a small pattern size and do not encode any information on the relative position of the patterns, the closest compound to **2a** is **1b**.

**Figure 1:**
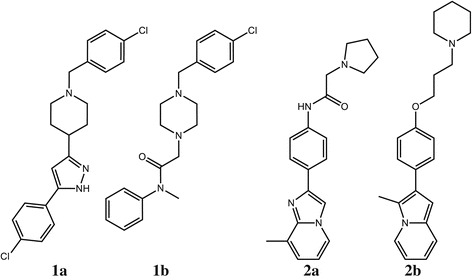
**Example molecules (from Reference [** 15 **]).** The similarity matrix with all pairwise similarities of these compounds with various metrics is given in Table S1 in the Additional file 1.

Graph based similarity methods such as Maximum Common Substructure (MCSS) [12,18] and Maximum Overlapping Set (MOS) [15] similarity based methods put a high weight on the common substructure. The similarity is computed from the ratio of the number of atoms and bonds in the MCSS or MOS to the total number of atoms and bonds. Because the similarity calculation is based on the integer counts, it is not sensitive to differences in substitution patterns. Stahl *et al*. introduced two empirical correction terms to mitigate this problem: one to penalize different arrangements of common substructures and the second to increase the similarity value if the two molecules have a substructure in common that exceeds 70% of the smaller molecule [15]. When comparing the compounds in Figure 1 the pairs **1a/1b** and **2a/2b** constitute nearest neighbors in agreement with chemical intuition.

For clustering fragment hits we have developed a new similarity metric: the Atom-Atom-Path (AAP) similarity metric that does not need empirical terms. AAP, too, puts a high weight on the common substructures. In the case of the compounds in Figure 1 it correctly recognizes the pairs **1a/1b** and **2a/2b** as nearest neighbors. The calculation of the AAP similarity of two molecules A and B consists of four steps. In the first step, each atom is described by the set of linear paths originating from it. In the second step, the atom-to-atom similarity of each atom in A to each atom in B is computed. This similarity matrix is used in the third step to map each atom in the smaller molecule to a unique atom in the other. The final step consists of computing the AAP similarity from the atom-to-atom similarities of the mapped atom pairs.

Because the set of atom paths encodes the neighborhood of the given atom, the more similar the sets of paths of two atoms are, the larger the atom-to-atom similarity is. An atom-to-atom similarity of 1 indicates that the two atoms are embedded in a large substructure present in both molecules. As a result, the AAP similarity of a molecule pair increases with the size of the common substructures. The change of a single atom affects the atom-to-atom similarities of neighboring atoms. The AAP similarity is sensitive to very small changes in substitution. Hence, we found that molecules with AAP similarities as low as 0.3 still share many common features. The similarity distribution of AAP as well as circular and linear fingerprint similarities for the full pairwise comparison of 4000 compounds from the Novartis-GNF Malaria Box data [19] sets is shown in Additional file 1: Figure S3. The distributions show how the AAP similarities are generally much lower than the fingerprint based similarities.

Similarity for use to organize chemical compounds for medicinal chemists is highly subjective [20]. During repeated use at Genentech we have found that clustering using AAP similarity consistently gives results that are in agreement with medicinal chemists’ perception. The mapping of atoms ensures that each atom is considered equally and the description of the atoms ensures a fine grained weighting through their environment. This results in clusters in which ring systems have a high weight avoiding the formation of clusters with large differences in scaffolds. To provide a comparison with Tanimoto similarity using linear and circular fingerprints we have included the clustering of a random subset of the Novartis-GNF Malaria Box dataset using three similarity metrics as Additional file 2.

## Implementation

**Figure 2:**
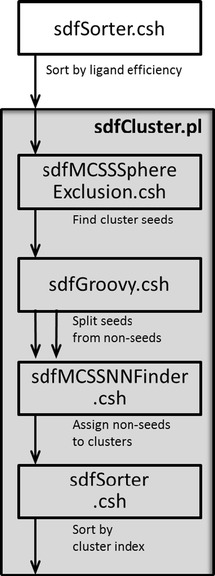
**Simplified workflow of clustering application.** Each box represents a command line program.

All programs required to cluster molecules using the DISE method and the AAP similarity are available in Additional file 3. Clustering using the DISE algorithm is performed by applying two command line programs to the input data, i.e. sdfSorter.csh and sdfCluster.pl (Figure 2). sdfSorter.csh sorts records in SDF files by the value of one or more fields that must be present in the records. sdfCluster.pl performs the SE clustering by internally executing the sequence of command line programs shown in Figure 2. The programs sdfMCSSSphereExclusion.csh and sdfMCSSNNFinder.csh use the AAP similarity for finding the cluster seeds and for assigning the cluster members to the closest seed. Below we describe the algorithm to compute the AAP similarity.

### Atom-Atom-Path similarity algorithm

Each atom is represented by a list of linear paths originating from all bonds on the given atom and extending up to 7 bonds. These linear paths are computed using a depth first algorithm. A path is encoded as a sequence of integer pairs (b_k_,a_k_). b_k_ is the integer representing the bond type of the k^th^ bond (1: single, 2: double, 3: triple, 4: aromatic). a_k_ is the integer representing the atom type of the k^th^ atom. For aliphatic atoms, a_k_ equals the atomic number. For aromatic atoms 108 is added to the atomic number. The path can then be stored as a unique integer p according to the Equation 1:

1 $$ \mathrm{p}=\left(\left(\left(\left({\mathrm{b}}_1*\mathrm{n}\mathrm{A}\mathrm{T} + {\mathrm{a}}_1\right)*\mathrm{n}\mathrm{B}\mathrm{T}+{\mathrm{b}}_2\right)*\mathrm{n}\mathrm{A}\mathrm{T}+{\mathrm{a}}_2\right)*\mathrm{n}\mathrm{B}\mathrm{T}+\dots .\right) $$

nAT is the number of atom types (2x108) plus one and nBT is the number of bond types (4) plus one. This representation guarantees a unique integer for each possible path. In the current implementation the path is stored as unsigned integer number with 16 bits. Overflows during the computation of p are ignored and might result in collisions. We have compared similarities calculated using p stored in 8, 16, 32 and 64 bit integer numbers and found that using 16 bit integers gives very similar results to using 64 bits. Using 16 bit integers has a slight performance benefit. If a path is found multiple times, when performing the depth first search on an atom, it is stored multiple times in the list of paths.

Given the list of paths on each atom in A and B, the similarity Sim_Ai,Bj_ of the two atoms A_i_ and B_j_ is computed using Equation 2.

2 $$ \mathrm{S}\mathrm{i}{\mathrm{m}}_{Ai,Bj}={\delta}_{Ai,Bj}\frac{nc_{Ai,Bj}+{\delta}_{Ai,Bj}}{Max\left({np}_{Ai},{np}_{Bj}\right)*2-{nc}_{Ai,Bj}+{\delta}_{Ai,Bj}} $$

**Figure 3:**
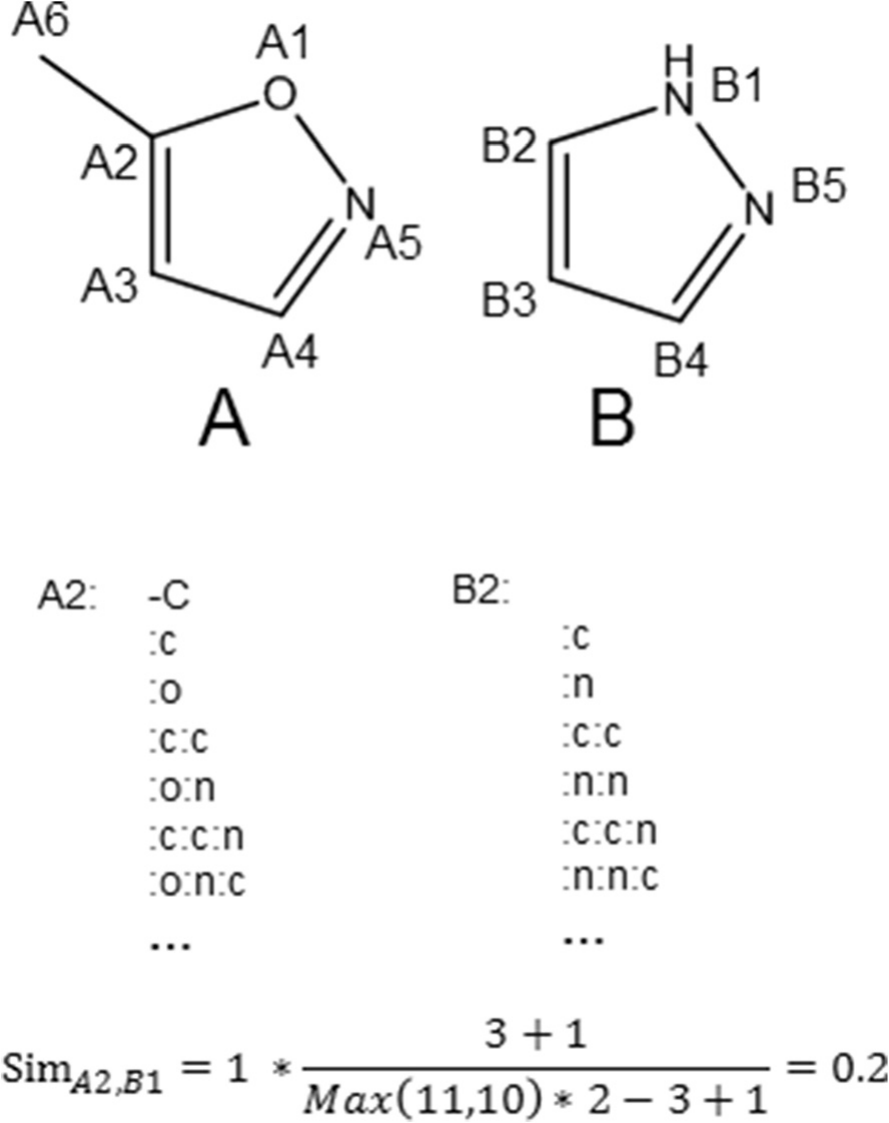
**Computation of the atom-to-atom similarity between atoms A2 and B2.** Paths originating at atoms **A2** and **B2** are listed. Upper case letters denote aliphatic atoms; lower case letters denote aromatic atoms. Columns (:) denote aromatic bonds. Hyphens (−) denote single bonds.

δ_Ai,Bj_ is 1 if Ai and Bj are of the same atom type and 0 if they are not. nc_Ai,Bj_ denotes the number of paths that atom Ai in molecule A and atom Bj in molecule B have in common. np_x_ denotes the total number of paths originating from atom x. Other equations for computing the atom and molecular similarities were evaluated but were found to be less suitable (cf. Additional file 1). An example calculation is shown in Figure 3.

**Figure 4:**
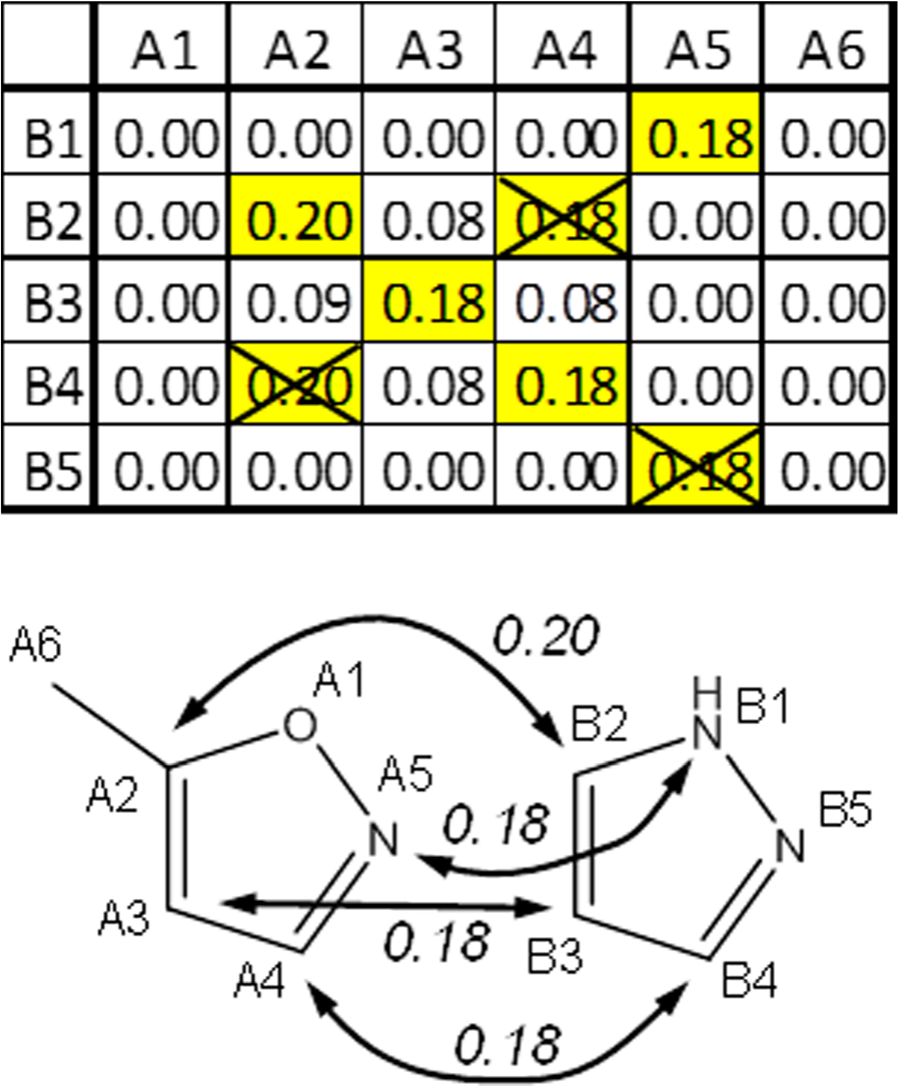
**Alignment of the atoms in molecules A and B based on the atom-to-atom similarity matrix.** Highlighted atom pairs mark the highest similarity mapping for each atom. Some atom pairs are crossed out in the matrix to prevent the mapping of one atom in molecule B to multiple atoms in molecule A. Note that the pairs of atoms B1/B5 and B2/B4 are considered as symmetry equivalent as all bonds in B are aromatic and hydrogen atoms are not considered. A5 was assigned to B1 and not B5 because of the order of the atoms in the molecule file.

The next step of the AAP similarity computation is the mapping of atoms in molecules A and B. The goal is to map each atom in the smaller molecule onto one atom of the larger molecule while maximizing the sum of the atom-to-atom similarities ΣSim_Ai,Bj_. It is illustrated in Figure 4. This problem is well known in Operation Research as the Assignment Problem and the Hungarian Algorithm was developed to efficiently solve this problem [21]. We have implemented the mapping step using the Hungarian Algorithm as provided in Java by Nedas [22]. However, we found that a heuristic algorithm significantly improves the performance while yielding results that only infrequently deviate from the optimal solution. When deviations are found, the difference from the optimal solution is minimal (cf. Additional file 1: Figure S1). The heuristic algorithm was implemented as follows: First, the two atoms with the highest Sim_Ai,Bj,_ in the full similarity matrix are mapped to each other. These two atoms are removed from the similarity matrix and the first step is repeated until all atoms have been assigned. In case of ties, the mapping is performed in the order of the atoms in the input molecules. All results in this paper were computed using the heuristic algorithm unless otherwise noted. Optimal Assignment Kernels follow a similar concept and have been used in machine learning methods such as Support Vector Machines [23].

The final step is the computation of the AAP similarity using the following equation:

3 $$ \mathrm{S}\mathrm{i}{\mathrm{m}}_{A,B}=\frac{\sum_{Mapped\  Atoms\ \left(i,j\right)}{Sim}_{Ai,Bj}}{Max\left({na}_A,{na}_B\right)*2-{\sum}_{Mapped\  Atoms\ \left(i,j\right)}{Sim}_{Ai,Bj}} $$

The summation is performed over the pairs of mapped atoms. na_A_ and na_B_ denote the number of atoms in molecules A and B. The similarity for the two example molecules in Figure 3 is calculated as follows:

$$ Si{m}_{A,B}=\frac{0.2+0.18+0.18+0.18+0}{Max\left(6,5\right)*2-0.74}=0.066 $$

The range of Sim_A,B_ is between 0 and 1. Given two identical molecules A and A’, all atoms will be mapped to equivalent atoms yielding atom-to-atom similarities Sim_Ai,A’i_ equal to 1. Therefore, the sum over the mapped atoms in Equation 3 will yield the number of atoms and as a result Sim_A,A’_ will be 1. Any difference between structure A and B will yield atom-to-atom similarities which are lower than 1, therefore Sim_A,B_ will be less than 1.

Run times for the AAP similarity calculations are significantly longer than for computing similarities using the Tanimoto coefficient and binary fingerprints. Table 1 shows the run times for the computation of the N^2^ similarity matrix for three methods and three different numbers of compounds. For the fingerprint based computation 2048 bit linear fingerprints were pre-computed with in-house software. The fingerprint based similarity computation (FP) is by far the fastest, completing 16 million comparisons in 8 seconds. Using the AAP method takes longer. Computing the full similarity matrix of 4000 compounds, which is close to the size of larger fragment libraries, requires ca. 33 minutes. The same calculation is estimated to take approximately 6 days when using the MOS method [24]. Thus a fingerprint based method is still the method of choice for computing the similarity matrix for very big datasets.

**Table 1:** **Elapsed time for single threaded similarity matrix computation**

**Method**	**Number of compounds**	**Number of comparisons**	**Elapsed time**
FP	40	1.6 10^3^	0.5 s
FP	400	1.6 10^5^	0.7 s
FP	4000	1.6 10^7^	8 s
AAP	40	1.6 10^3^	1 s
AAP	400	1.6 10^5^	19 s
AAP	4000	1.6 10^7^	33 min
MOS	40	1.6 10^3^	62 s
MOS	400	1.6 10^5^	84 min
MOS	4000	1.6 10^7^	~6 days

Time for 4000 compounds with MOS is extrapolated.

### Implementation details

The code used to perform the DISE clustering with AAP similarity is available in the Additional file 3. The readme file in the root directory contains information on how to install, compile and get further documentation. The readme file in the “examples/NovartisMalariaBox” subdirectory explains how to reproduce the DISE clustering with AAP similarity using data from the Novartis-GNF Malaria Box [19].

All command line programs use the OpenEye toolkit [25] for reading and writing SDF files and accessing the fields in SDF files. Additionally, the OEChem API is used for traversing the molecular graph while computing the AAP similarity. Some of the command line programs used internally in sdfCluster.pl are part of the open source package Autocorrelator [26] that is available on GoogleCode (c.f. Table 2).

**Table 2:** **List of command line programs used in the clustering workflow**

**Program name**	**Source**	**Use**
sdfSorter.csh	Autocorrelator	sort input records by LE and sort output by cluster index
sdfGroovy.csh	Autocorrelator	modify SDF fields and filter records by field contents
sdfTagTool.csh	Autocorrelator	copy, rename and delete SDF fields
sdfMCSSSphereExclusion.csh	Additional file 3	select cluster seeds
sdfMCSSNNFinder.csh	Additional file 3	assign cluster members to seeds
sdfCluster.pl	Additional file 3	perform sphere exclusion clustering by calling other tools (cf. Figure 2)

## Results and discussion

Utilizing additional fragment collections, we expanded our previous ~2,500 compound screen of the MAP4K4 kinase [27] by performing an additional screen of 8,000 compounds [28]. Approximately 600 additional hits with LE ranging from 0.24 to 0.67 and with K_D_’s between 3 and 930 μM were identified and added to the earlier hit set (Note: LE was computed as 1.4*pK_d_/nHeavyAtoms). AAP similarity and DISE clustering were used to cluster the combined hit set fragments. The fragment hits were sorted according to their LE, followed by the cluster seed selection. The AAP similarity threshold in this step was 0.3. The remaining hits were assigned to the most similar cluster seed.

**Figure 5:**
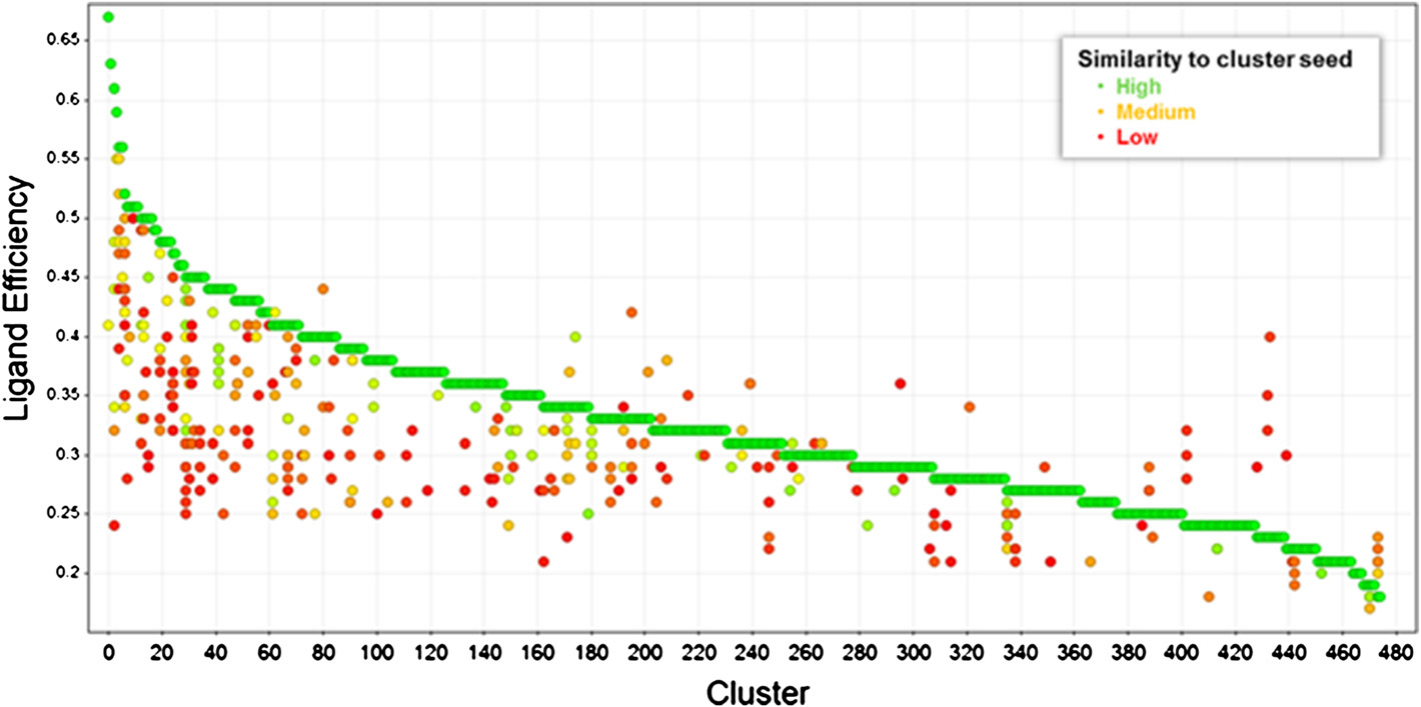
**Scatterplot of the fragment hits by LE over cluster index.**

The DISE clustering results can be visualized in a LE-over-Cluster plot as shown in Figure 5. Each vertical set of points in this plot represents one cluster. The points are colored by similarity of the given cluster member to the respective seed (green = high similarity, red = low similarity). The LE values are in the range of 0.2 to 0.67 with K_D_ values from 870 nM to 3.4 mM. As expected a monotonically decreasing curve of green points is formed by the cluster seeds. Most cluster members have lower LE than their respective seeds and, hence, appear below the curve. The cluster members that appear above the curve are located in the overlapping region of multiple spheres, i.e. they are in the vicinity of more than one seed, and were assigned to the most similar seed. Most of them have relatively low similarities to their respective seeds. It can be seen that many of the cluster members with high similarity to the cluster seed (colored in green) are vertically close to the seed. These compounds exemplify the *similarity principle*, [29] i.e. similar compounds have similar binding affinity. The principle also applies to LE because LE is directly proportional to the binding affinity and the range of heavy atom counts is narrow within a cluster.

The plot can be used to identify clusters containing some initial SAR. Green points that differ largely in LE from their cluster seed are indicative of activity cliffs because small structural changes strongly affect the LE. Red points that differ little in LE from the seed may suggest structural changes that affect the ligand binding mode. These compounds may indicate scaffolds that are prone to flipping their binding pose or selecting different protein conformations during hit expansion.

**Figure 6:**
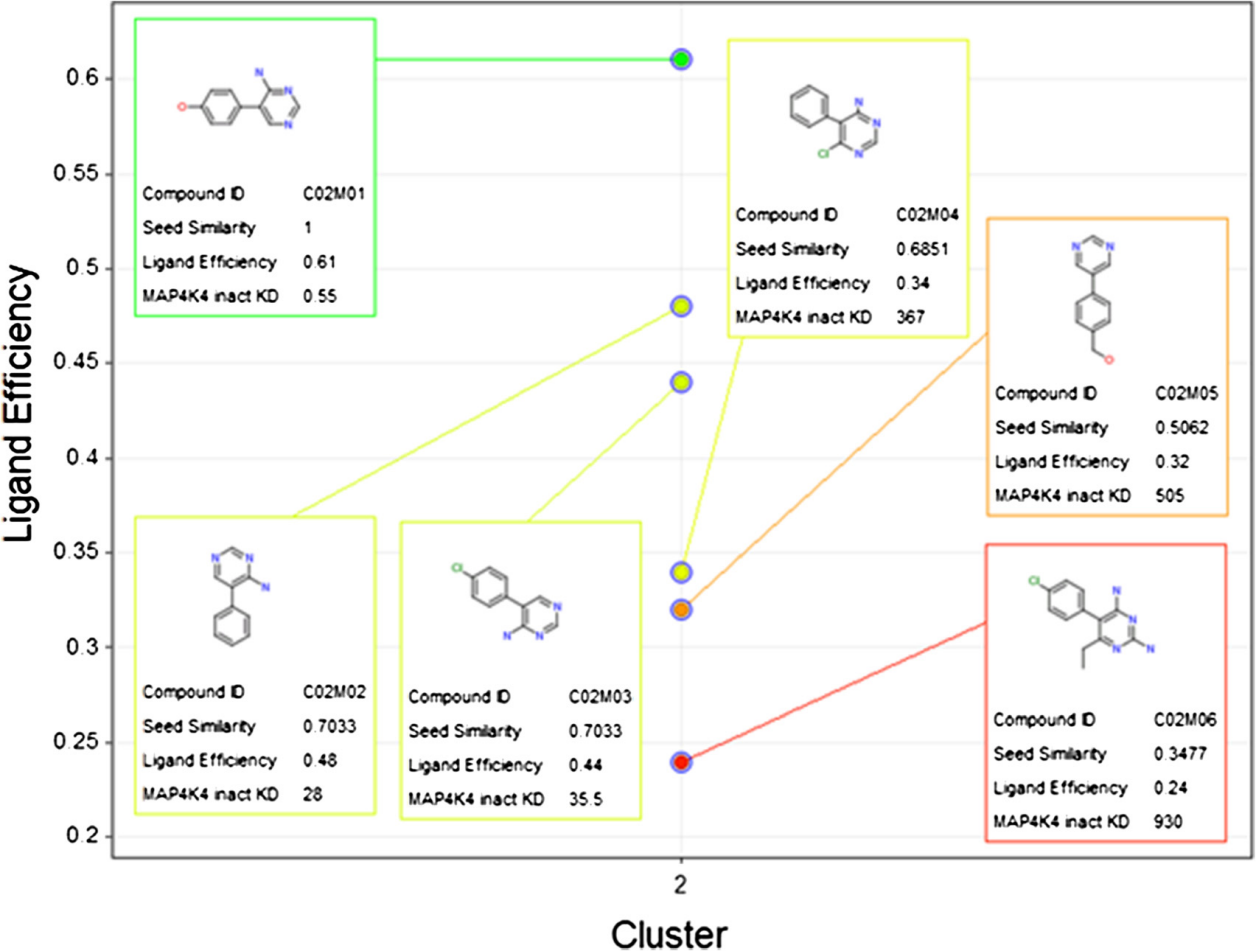
**Cluster 2 showcases the sensitivity of the AAP similarity to minimal differences in the molecules.** The effect of these differences on the LE reveals SAR. K_D_ values are in μM.

**Figure 7:**
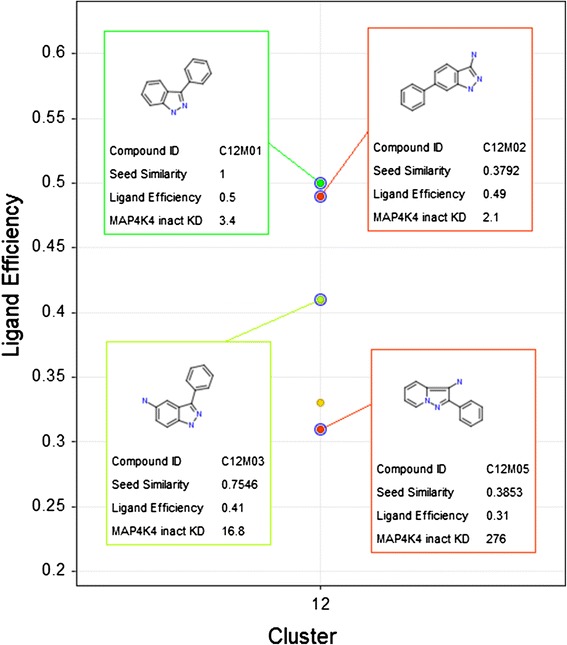
**Cluster 12 showcases the effect of changing the position of the phenyl substituent on the indazole ring.** K_D_ values are in μM.

Cluster 2 and 12 are good examples to showcase the characteristics of DISE clustering using AAP similarity (Figures 6 and 7).

- The cluster seed may not be the smallest molecule in the cluster as exemplified by C02M01 and C02M02. This is intentional as the C02M01 has the higher LE.
- Equivalent structural transformations result in identical similarities. C02M02 and C02M03 differ in replacement of the oxygen by hydrogen and chlorine, respectively. They have the same similarity of 0.70 to the cluster seed.
- The AAP similarity is sensitive to the substitution pattern. C02M03 and C02M04 differ only in the position of the chlorine atom. In C02M03 the chlorine is a direct replacement for the oxygen in the cluster seed C02M01 while C02M04 differs from the seed in two positions. This results in lower similarity for C02M04. If even more atoms in the molecules are changed, as in C02M05 and C02M06, then the similarity drops further.
- A cluster can contain molecules with differences in the core ring system. C12M02 differs from the seed C12M01 in the position of the phenyl group. C12M05 contains a pyrazolo pyridine ring instead of the indazole ring. Both, C12M02 and C12M05 have low similarity to the cluster seed, 0.38 and 0.39 respectively. These values are close to the sphere exclusion threshold of 0.3.

A few activity cliffs can be immediately identified in these clusters. C02M02 and C02M03 show a LE difference of 0.13 and 0.17 to the cluster seed C02M01 although there is a single atom change. C12M03 has also a single atom change to its cluster seed and exhibits a 0.09 difference in LE. Another interesting SAR finding is the small difference in LE between C12M01 and C12M02 although they differ in the core ring system. This is indicative of a change in binding mode or could be due to the phenyl ring in C12M02 occupying a new high affinity binding pocket.

**Figure 8:**
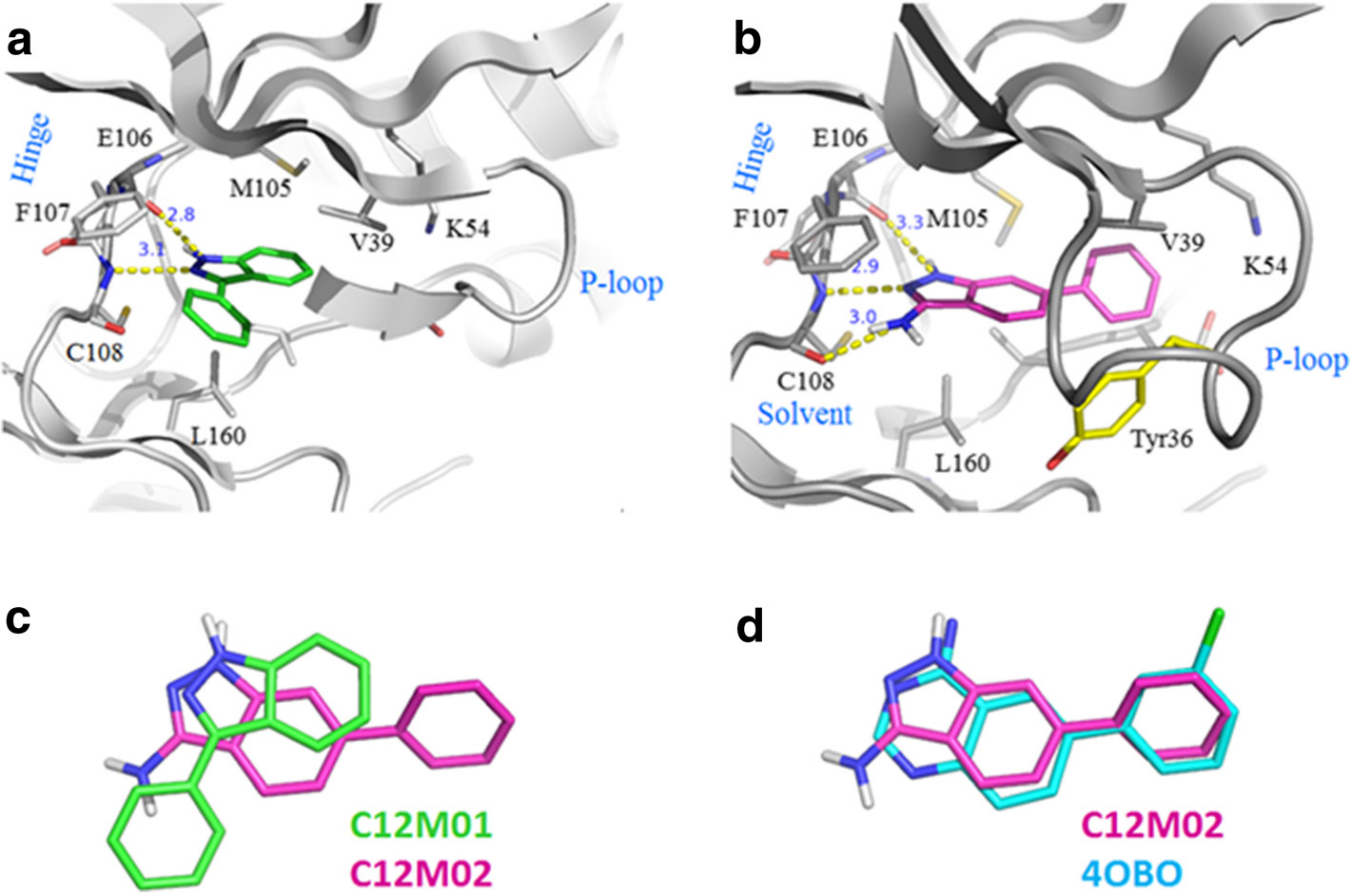
**Docking results. (a)** Model of C12M01 bound in the MAP4K4 binding site with extended P-loop conformation. **(b)** Model of C12M02 bound in the pocket with folded P-loop conformation. **(c)** Overlay of docking poses of C12M01 and C12M02. **(d)** Overlay of the docking pose of C12M02 and the crystallographic pose of an amino quinazoline ligand in 4OBO.

MAP4K4 co-crystal structures were not determined for these two molecules. However, using well established docking protocols [27,30] to dock the two molecules into the ATP binding site of two different MAP4K4 crystal structures suggests different binding modes. The P-loop in these two crystal structures has either an extended or a folded conformation. It is very intriguing that C12M01 selectively docks to the P-loop extended conformation (Figure 8a, Glide docking score = −9.0), while C12M02 selectively docks to the P-loop folded conformation (Figure 8b, Glide docking score = −9.7). The common substructures of C12M01 and C12M02 in the docking models both interact with the hinge region of MAP4K4 but are rotated slightly to avoid steric clashes and also to gain maximum protein-ligand interactions with the respective proteins (Figure 8c). The phenyl rings attached to the indazole of these two compounds occupy different binding pockets and therefore make different interactions with the protein. Another key difference is that the indazole core in C12M02 forms additional van der Waals and edge to face interactions to the TYR36 residue of the folded P-loop. Docking cannot be considered conclusive for the binding mode. However, the overlay of C12M02 with an amino quinazoline MAP4K4 inhibitor that binds to the folded P-loop conformation shows how similar the suggested binding mode of C12M02 is to the binding mode of the amino quinazoline lead (Figure 8d, [27], PDB ID: 4OBO). We attribute the nearly equivalent LE of C12M01 and C12M02 to a combined effect of filling different pockets and gaining additional interactions through protein conformational changes.

Most screening campaigns produce too many hits to pursue with structure determination or chemical follow-up and the list must be reduced to a small number of high priority compounds. The structural features and physico chemical properties that make a fragment attractive to a given medicinal chemistry program will vary by project. For fragments the LE parameter is typically given a significant weight. During the fragment hit triage meeting, each hit is considered for follow-on activities, including crystallography and SAR exploration through purchase or limited synthesis of analogs. Fragments of high priority are assigned a score of 1, fragments of moderate priority and backups for priority 1 compounds are assigned a score of 2, and hits not of interest a score of 3. When reviewing hit sets clustered using undirected methods every single row must be discussed as the highest LE molecules may be anywhere in the list. For large sets, this can take several hours resulting in fatigue and unintentional exclusion of attractive compounds that fall late in the list. The MAP4K4 screen, as with most of our screens, returned a hit set with 30-50% of the members having LE < 0.3. We could have accelerated the triage by removing them from the hit set. However, when using DISE with AAP similarity the low LE members of high LE clusters stay grouped together giving a more comprehensive view of the SAR as described above, and increased confidence in the chemotypes. This leaves fewer, but more complete, clusters of high interest for review.

The results of the clustering of the MAP4K4 hits were presented to the panel of experts in the order of the cluster index. The panel was asked to collaboratively and interactively assign a score to each hit. To facilitate the conversation the entire hit set was pre-scored on the 1–3 scale using a simple binning and linear weighting function. Briefly, the LE, potencies, chemical diversity (based on the DISE results), selectivity against other co-screened kinases and our historical kinase fragment screening data, historical crystallization success on different targets, were grouped into high, medium, or low bins based on histograms for each property for the full hit set. The linear weighting approach means that different or additional properties can also be included as suits a particular project, such as competition assessments, NMR binding data, thermal melt or enzyme inhibition data, etc. Points were assigned for each property (2 points for a high bin, 1 for a medium, and zero for a low bin). Scores were added and binned by histogram into score 1, 2, and 3. These computed scores were included in the data sheets as unbiased assessments of the compound properties to help focus the discussion towards the merits of the chemistry more than potency. The time for the prioritization meeting was reduced by half. The team submitted 61 compounds to the X-ray workflow providing valuable information for the MAP4K4 project team [27].

## Conclusions

We have described the implementation and use of AAP similarity coupled with DISE clustering as a tool to organize fragment hits. The presorting of the hits by decreasing LE ensures that the most interesting clusters appear at the top, thus, drawing the attention of the review team to the most promising compounds. While we have used LE in the discussion here, in principle the dataset can be presorted and re-clustered based on other properties or efficiency indices. The detailed description of atom neighborhoods used in the AAP similarity allows for the differentiation of even small changes in molecular structure. The combination of the AAP similarity and DISE clustering has also been successfully applied to prioritize high throughput screening hits [31]. The code for this software is available in Additional file 3.

## Availability and requirements

**Availability:** As zip file including source code and examples in the Additional file 3.

**Operating system(s):** Platform independent (tested on Linux).

**Programming language:** Java, (csh and perl wrappers around the java programs).

**Other requirements:** Java 1.6 or higher; Command line programs from the Autocorrelator open source project; OEChem Toolkit 2013 (commercial license required).

**License:** Apache License.

**Any restrictions:** software requires license for OEChem Toolkit (commercial license required).

## Additional files

Additional file 1:
**AAPathClust_supl.pdf. Figure S1**: Visualization of differences between AAP similarity computed using atom mapping with Hungarian and heuristic algorithm. Description of other similarity equations that were evaluated in addition to the Equations 2 and 3 in the paper. **Figure S3**: distribution of similarity pairs computed with AAP Similarity and Tanimto Similarity using linear and circular fingerprints. **Table S1**: pairwise similarities of compounds **1a**, **1b**, **2a** and **2b**. Details of clustering results in 4000.Clusters.tab.gz. Additional file 2:
**4000.Clusters.tab.gz.** Compressed file containing clustering results computed with AAP similarity and Tanimto similarity using linear and circular fingerprints. Additional file 3:
**gCheminfoCommands_20150126.tgz.** Zip file containing source code and compiled code for software described in this paper. Unpack the zip file into a directory on a UNIX server and follow the instructions in the = readme.md file to install the software. Step by step instructions on how to reproduce the clustering method on the Novartis-GNF Malaria Box dataset can be found in the examples/NovartisMalariaBox/=readme.md file.
