# Supplementary material for: Atom-Atom-Path similarity and Sphere Exclusion clustering: tools for prioritizing fragment hits
Source: J Cheminform. 2015 Mar 25;7:11. doi: 10.1186/s13321-015-0056-8 (PMC4392110; doi:10.1186/s13321-015-0056-8)
Supplement: Additional file 1: — AAPathClust_supl.pdf. Figure S1: Visualization of differences between AAP similarity computed using atom mapping with Hungarian and heuristic algorithm. Description of other similarity equations that were evaluated in addition to the Equations 2 and 3 in the paper. Figure S3: distribution of similarity pairs computed with AAP Similarity and Tanimto Similarity using linear and circular fingerprints. Table S1: pairwise similarities of compounds 1a, 1b, 2a and 2b. Details of clustering results in 4000.Clusters.tab.gz. [file 13321_2015_56_MOESM1_ESM.pdf]

Supplemental material

Comparison of atom mapping using Hungarian and heuristic algorithm

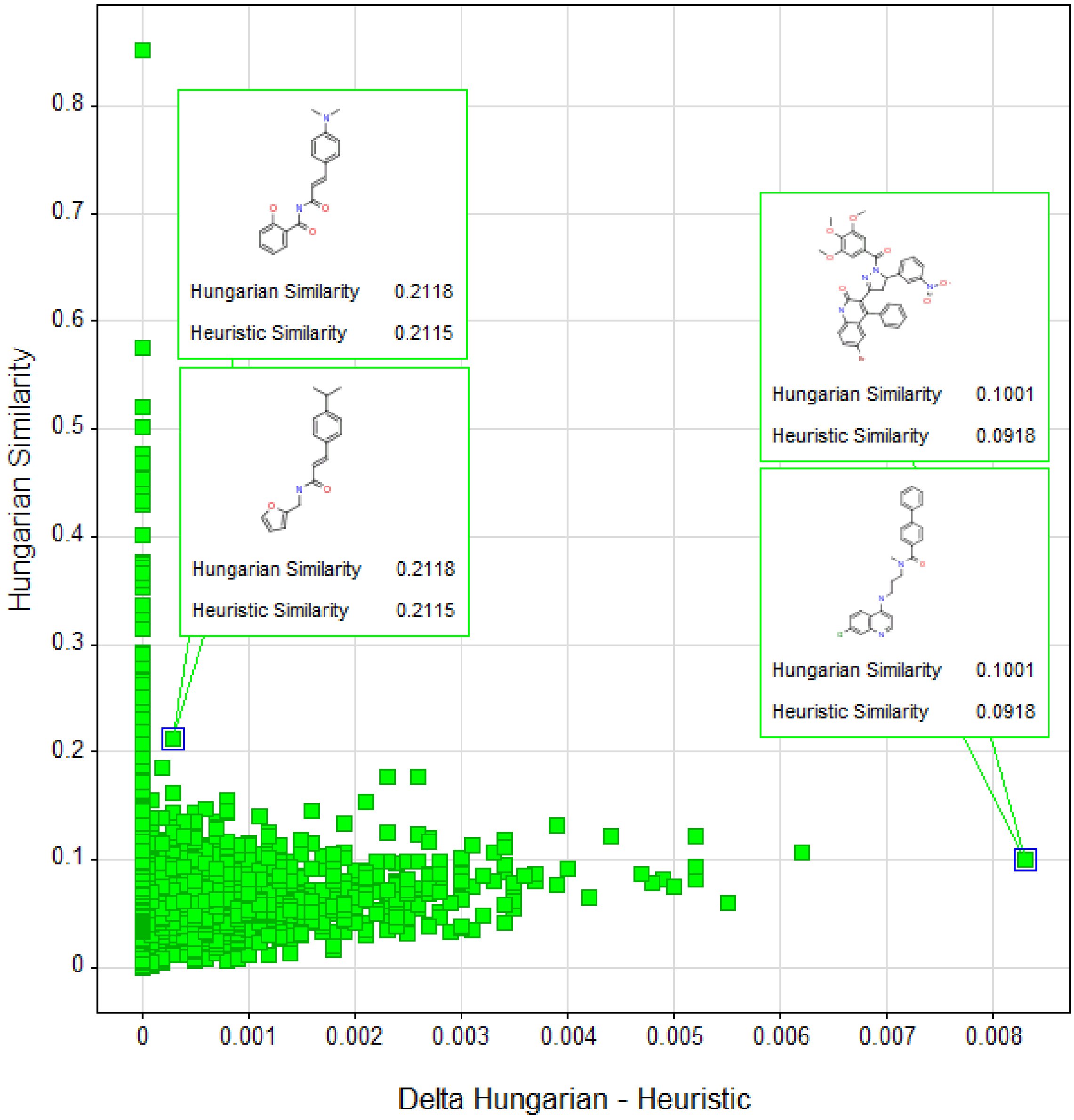

Figure S1. Comparison of similarities computed using the Hungarian and the heuristic algorithm for mapping atoms. The plot compares the similarity computed using the Hungarian algorithm to the difference found when using the heuristic method described in the paper. The x axis plots ΔSim = Sim(Hungarian) - Sim(Heuristic). The computation was done by computing all pairwise similarities of 101 randomly chosen molecules from the Novartis-GNF dataset to each other (10100 pairs). For 4763 pairs both algorithms yield identical results. The heuristic algorithm underestimates the similarity by no more than 0.0083. All pairs for which the algorithms differ had similarities below 0.22, which is below the clustering threshold of 0.3 used for the fragments screening hits.

Equations for computing atom pair and molecular similarities:

The following formulas were evaluated (all abbreviations are as described in the main article):

Atom pair similarity: $Sim_{Ai,Bj}$

$$\delta_{Ai,Bj} \frac{nc_{Ai,Bj} + \delta_{Ai,Bj}}{Max(np_{Ai}, np_{Bj}) * 2 - nc_{Ai,Bj} + \delta_{Ai,Bj}} \quad \textbf{(2)}$$

$$\delta_{Ai,Bj} \frac{nc_{Ai,Bj} + \delta_{Ai,Bj}}{np_{Ai} + np_{Bj} - nc_{Ai,Bj} + \delta_{Ai,Bj}} \quad \textbf{(2b)}$$

$$\delta_{Ai,Bj} \frac{nc_{Ai,Bj} + \delta_{Ai,Bj}}{Max(np_{Ai}, np_{Bj}) + \delta_{Ai,Bj}} \quad \textbf{(2c)}$$

Molecular similarity: $Sim_{A,B}$

$$\frac{\sum_{Mapped\ Atoms\ (i,j)} Sim_{Ai,Bj}}{Max(na_A, na_B) * 2 - \sum_{Mapped\ Atoms\ (i,j)} Sim_{Ai,Bj}} \quad \textbf{(3)}$$

$$\frac{\sum_{Mapped\ Atoms\ (i,j)} Sim_{Ai,Bj}}{na_A + na_B - \sum_{Mapped\ Atoms\ (i,j)} Sim_{Ai,Bj}} \quad \textbf{(3b)}$$

$$\frac{\sum_{Mapped\ Atoms\ (i,j)} Sim_{Ai,Bj}}{Max(na_A, na_B)} \quad \textbf{(3c)}$$

All Equations yield similarities that range between 1 (identical atoms/structures) and 0 (no similarity). Initially Equation **2b** and **3b** were used. They are derived from the Tanimoto coefficient which is widely used for computing similarities of molecular fingerprints. However, it was found that this leads to higher similarities than expected when comparing molecules of different sizes or

branching as shown in Figure **S2**. Structure S1 is more similar to S3 (0.133) than S2 (0.113) using Equation **2b** and **3b**. This is counter intuitive as S1 and S2 differ only in the central atom. The relationship is reversed using Equation **2** and **3** or **2c** and **3c**.

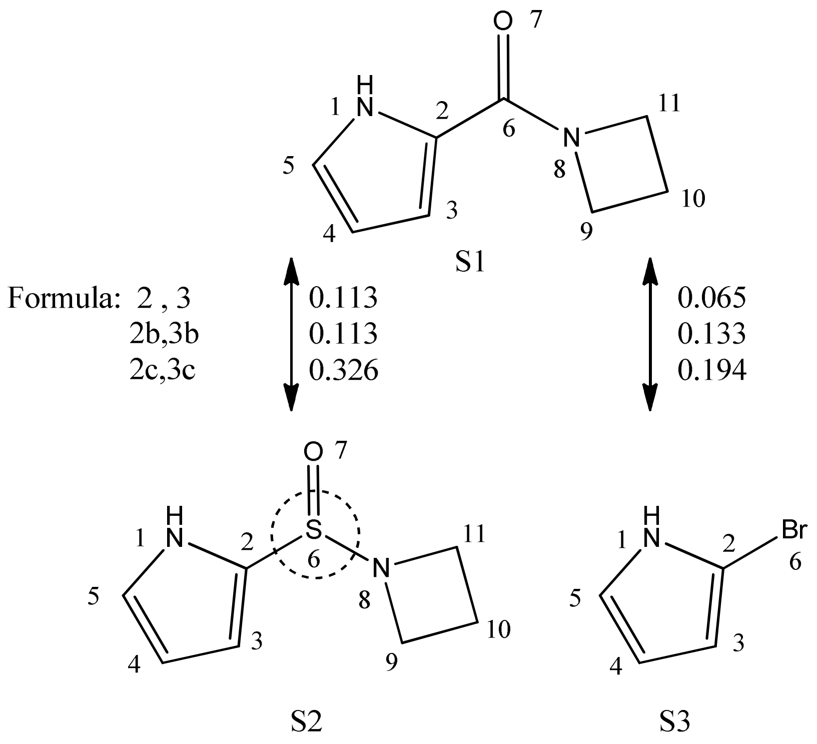

Figure S2. Examples of computing Atom-Atom-Path similarities using Equations **2** and **3**, **2b** and **3b** or **2c** and **3c**.

The mapping of the atoms is as expected using any of the Equations **2**, **2b** or **2c**. The mapping of molecule S1 onto S2 contains more atoms than the mapping of S1 onto S3. The individual atom to atom similarities are larger for S1,S3 when using Equation **2b** than when using Equation **2**. This is due to the difference in the number of paths (np) which pass through the modified atom #6. Each path passing through the modified atom decreases the similarity. However structures S1 and S2 have a higher number of paths going through atom #6 than structure S3 due to the larger and branched substitution in S1 and S2. Therefor the sum of paths term (np_{Ai} + np_{Bi}) in Equation **2b** is larger when comparing S1 to S2 than when comparing S1 to S3 resulting in $Sim_{Ai,Bi}$(S1,S2) < $Sim_{Ai,Bi}$(S1,S3).

By using the max() of the number of path in Equation **2** or **2c** the denominator is only dependent on the maximum number of path in both structures and therefore the similarity does not depend on the relative size of the substitutions as it does using Equation **2b**.

When combining the atom pair similarities to the whole molecule similarity using Equation **3b** the term computing the sum of atom counts (na_A + na_B) is larger for the S1,S2 comparison (11+11=22) than for the S1,S3 comparison (11+6=17). This also results in a lower similarity for Sim(S1,S2) than Sim(S1,S3) even if the similarity of each mapped atom pair were equal. This dependence on the size of the molecules can be removed by using the max() of the number of atoms as in Equation **3** or **3c**.

We decided to use the Equations **2** and **3** over **2c** and **3c** because Equations **2** and **3** yield a larger separation of highly similar compounds while compressing the range of dissimilar compounds. The combined effect is that Atom-Atom-Path similarity values are smaller than similarities computed using fingerprints. Compounds with similarities above 0.3 generally share a significant amount of features.

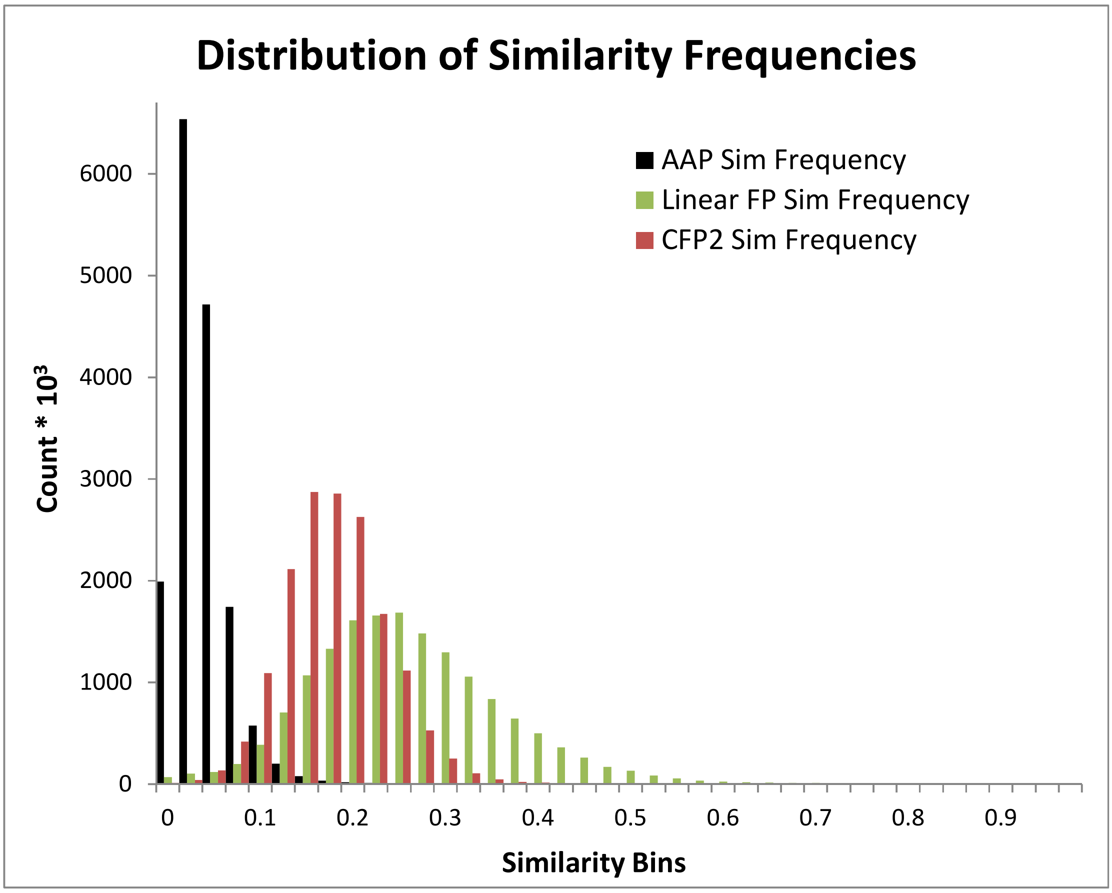

Figure S3. Distribution of similarities computed with AAP Similarity and Tanimto Similarity using linear and circular fingerprints. The graph summarizes 16 million comparisons for all pairwise comparisons of 4000 compounds chosen randomly from the Novartis-GNF Malaria Box dataset. Fingerprints and similarities were computed using in-house developed software. Linear fingerprints are of length 7 (comparable to daylight fingerprints) and circular fingerprints are of radius 2 (comparable to ECFP4).

Table S1. Similarities of example compounds from Figure **1**. The MOS similarity was computed using Spinifex [30] with default options. The other similarities were computed as described for Figure **S3**. For each group of four compounds with the same compound A the nearest neighbor is highlighted in bold.

Compounds		Similarity(A,B)			
A	B	Linear	Circular	MOS	AAP
**1a**	**1a**	1.00	1.00	1.00	1.00
**1a**	**1b**	0.21	**0.37**	**0.59**	**0.19**
**1a**	**2a**	0.17	0.18	0.28	0.06
**1a**	**2b**	**0.25**	0.20	0.34	0.09
**1b**	**1a**	0.21	**0.37**	**0.59**	**0.19**
**1b**	**1b**	1.00	1.00	1.00	1.00
**1b**	**2a**	**0.24**	0.27	0.22	0.12
**1b**	**2b**	0.10	0.21	0.18	0.05
**2a**	**1a**	0.17	0.18	0.28	0.06
**2a**	**1b**	0.24	**0.27**	0.22	0.12
**2a**	**2a**	1.00	1.00	1.00	1.00
**2a**	**2b**	**0.27**	0.26	**0.61**	**0.15**
**2b**	**1a**	0.25	0.20	0.34	0.09
**2b**	**1b**	0.10	0.21	0.18	0.05
**2b**	**2a**	**0.27**	**0.26**	**0.61**	**0.15**
**2b**	**2b**	1.00	1.00	1.00	1.00

Clustering of 4000 random compounds from Novartis-GNF Malaria Box dataset:

This dataset is provided for readers to compare clustering performed using different methods. 4000 compounds were picked randomly from the Novartis-GNF Malaria Box dataset. The file was sorted by "PF proliferation inhibition 3D7 EC50 uM" and the compounds were clustered using the AAP similarity with a similarity threshold of 0.3 as described in this paper. Linear fingerprints of path length 7 and circular fingerprints with radius 2 were added to the sdf file using in-house tools and the compounds were clustered using Tanimoto similarity on linear and circular fingerprints. The similarity

threshold was adjusted to yield a similar number of cluster seeds. The final seed count is between 1449 and 1450. The results of the three clustering methods were merged and saved as a tab separated file with the fields given in Table **S2**. The compressed tab separated file is part of the supplemental material (4000.Clusters.tab.gz).

Table S2. Fields in 4000.Clusters.tab.gz

Similarity Metric	Field Name	Description
	JC_XSMILES	SMILES as given in the original dataset
	PF proliferation inhibition 3D7 EC50 uM	EC50 as given in the original dataset
	GNF Pf identifier	Identifier as given in original dataset
AAP	AAP_clusterIdx	Index of cluster to which this compound was assigned
	AAP_centroidIdx	Indicates cluster seed with assigned cluster index
	AAP_ClusterSize	Size of cluster to which this compound was assigned
	AAP_NNSim	Similarity of this compound to cluster centroid
Tanimoto using Circular Fingerprints (radius 2)	CFP_clusterId	Index of cluster to which this compound was assigned
	CFP_centroidIdx	Indicates cluster seed with assigned cluster index
	CFP_ClusterSize	Size of cluster to which this compound was assigned
	CFP_NNSim	Similarity of this compound to cluster centroid
Tanimoto using Linear Fingerprints (path length 7)	LFP_clusterIdx	Index of cluster to which this compound was assigned
	LFP_centroidIdx	Indicates cluster seed with assigned cluster index
	LFP_ClusterSize	Size of cluster to which this compound was assigned
	LFP_NNSim	Similarity of this compound to cluster centroid
