# Supplementary material for: Atom-Atom-Path similarity and Sphere Exclusion clustering: tools for prioritizing fragment hits
Source: J Cheminform. 2015 Mar 25;7:11. doi: 10.1186/s13321-015-0056-8 (PMC4392110; doi:10.1186/s13321-015-0056-8)
Supplement: Additional file 3: — gCheminfoCommands_20150126.tgz. Zip file containing source code and compiled code for software described in this paper. Unpack the zip file into a directory on a UNIX server and follow the instructions in the = readme.md file to install the software. Step by step instructions on how to reproduce the clustering method on the Novartis-GNF Malaria Box dataset can be found in the examples/NovartisMalariaBox/=readme.md file. [file 13321_2015_56_MOESM3_ESM.zip › 13321_2015_56_MOESM3_ESM.tgz!/=readme.html]

# gCheminfoCommands (C) Genentech

The gCheminfoCommands software has been developed by the CDD Group at Genentech.
This software is released under the "Apache License Version 2.0".

gCheminfoCommands uses command line programs from the Open Source software
autocorrelator and the following libraries:

- Aestel\_20140613.jar
- commons-cli-1.2.jar
- groovy-all-1.8.8.jar
- jdom.jar
- oejava-2014.Feb.3-Linux-x64.jar (not included)
- testng-6.2.jar

## Licensing

Copies of the licenses can be found in the license subdirectory.

autocorrelator.jar

> The autocorrelator package is a separate open source package.
> The autocorrelator.jar is released under the
> GNU General Public License v3.
> The classes in autocorrelator.jar are not linked to any code in the
> gCheminfoCommands package.
> More information including the autocorrelator source code is available at:
> https://code.google.com/p/autocorrelator/

OEChem java library:

> *THE OPENEYE OECHEM JAR FILE IS NOT INCLUDED IN THIS PACKAGE!*   
> The oechem java library is required for most of the programs in this package.
> To obtain the library and a license to use it please contact:
> OpenEye Scientific software

All other files

> are released under the
> Apache License Version 2.0

## Installation on Linux

- Unpack the gCheminfoCommands\_XXX.tgz file
- Contact OpenEye
  for a copy of their oechem java toolkit.

  - copy the jar file from OpenEye into `gCheminfoCommands/lib` directory.   
    The examples were tested using `oejava-2014.Feb.3-Linux-x64.jar`
  - set the OE\_LICENSE environment variable to point to your
    OpenEye license file. e.g.   
    `setenv OE_LICENSE ~oechem/etc/oe_license.txt`
- Add the gCheminfoCommands/bin path to your unix path, e.g.   
  `set path=($path ~/gCheminfoCommands/bin)`
- Define the AESTEL\_DIR environment variable pointing to this directory e.g.   
  `setenv AESTEL_DIR ~/gCheminfoCommands`

## Compiling

In order to compile this package you must have installed the oechem java
toolkit as described under "Installation on Linux".
In addition you must have ant
installed and in your path.
Once these two conditions are met simply type `ant` in the root directory
of this package and the gCheminfoCommands jar file will be compiled.

## Creating JavaDoc

Issue `ant javaDoc`. The documentation will be created in the doc sub directory.

## Testing

- Regression tests:   
  Regression tests can be executed by issuing `ant test`
  At this time important low level methods have regression tests but many
  higher level methods do not.
- Functional tests:   
  A minimal functional test can be performed by issuing:
  `test/simple.csh`
  from the root directory of the package.
  This will execute the core command line programs and compare the resulting
  output to a reference file.

## Usage

Wrapper to run the Genentech command line tools and some of the autocorrelator
tools are located in the bin subdirectory. Each of the commands will print an
explanation of the command and the available options when executed without
any command line options.

Examples demonstrating the clustering process described in the paper can be
found in:   
`examples/NovartisMalariaBox/=readme.md`

## Other Operating Systems

The java code should run on other environments than linux as long as
the OpenEye library is available. However this has not been tested and
modified wrappers might be required.
The authors do not currently have experience with other environments
but would welcome reports describing the setup. For the time being this
package should be considered linux only.
