# Supplementary material for: Atom-Atom-Path similarity and Sphere Exclusion clustering: tools for prioritizing fragment hits
Source: J Cheminform. 2015 Mar 25;7:11. doi: 10.1186/s13321-015-0056-8 (PMC4392110; doi:10.1186/s13321-015-0056-8)
Supplement: Additional file 3: — gCheminfoCommands_20150126.tgz. Zip file containing source code and compiled code for software described in this paper. Unpack the zip file into a directory on a UNIX server and follow the instructions in the = readme.md file to install the software. Step by step instructions on how to reproduce the clustering method on the Novartis-GNF Malaria Box dataset can be found in the examples/NovartisMalariaBox/=readme.md file. [file 13321_2015_56_MOESM3_ESM.zip › 13321_2015_56_MOESM3_ESM.tgz!/examples/NovartisMalariaBox/=readme.html]

# NOTES

Any commands given in this readme file require the correct installation
of the gCheminfoCommands package and all settings described in the `=readme.txt`
located file in the root directory of the gCheminfoCommands package.

## Files in this directory

- Novartis\_GNF\_Assay\_Desc.doc, Novartis\_GNF.xls   
  Downloaded on 6/20/2014 from:

  - https://www.ebi.ac.uk/chemblntd/download/#tcams\_dataset
  - ftp://ftp.ebi.ac.uk/pub/databases/chembl/ChEMBLNTD/set2*gnf/Novartis*GNF.xls
  - ftp://ftp.ebi.ac.uk/pub/databases/chembl/ChEMBLNTD/set2*gnf/Novartis*GNF*Assay*Desc.doc
- Novartis\_GNF\_NoModifier.smi   
  Created by removing any rows with ">" in the "PF proliferation inhibition 3D7 EC50 uM" field
  in Novartis\_GNF.xls and saving the file as tab separated file.
- Novartis\_GNF\_NoModifier.sdf   
  created using:   
  `tab2Sdf.csh -in Novartis_GNF_NoModifier.smi -out Novartis_GNF_NoModifier.sdf`

## Citing the data (from: https://www.ebi.ac.uk/chemblntd)

> If you publish on, or wish to reference the Novartis-GNF dataset, please include the link to ChEMBL-NTD (www.ebi.ac.uk/chemblntd) and adapt the following citation language: Novartis-GNF Malaria Box, K Gagaring, R Borboa, C Francek, Z Chen, J Buenviaje, D Plouffe, E Winzeler, A Brinker, T Diagana, J Taylor, R Glynne, A Chatterjee, K Kuhen. Genomics Institute of the Novartis Research Foundation (GNF), 10675 John Jay Hopkins Drive, San Diego CA 92121, USA and Novartis Institute for Tropical Disease, 10 Biopolis Road, Chromos # 05-01, 138 670 Singapore

## Running the DISE algorithm with AtomAtomPath similarity and using `PF proliferation inhibition 3D7 EC50 uM` as sorting criterion

Execute the piped commands given below. Four command line programs are executed in sequence:

1. `sdfGroovy` this will remove any `<` characters in the `PF proliferation inhibition 3D7 EC50 uM` field.
2. `sdfSorter` sort sdf file ascending by `PF proliferation inhibition 3D7 EC50 uM`
3. cluster using `-AAPathSim DEFAULT` and a maximum similarity of 0.3 which
   corresponds to a sphere size of 0.7
4. compress the result with `gzip`   
   Finally the results are written into a file named:
   `Novartis_GNF_NoModifier.AAPath.0.3.sdf.gz`

The execution will take 10-20min depending on your hardware.
During the execution a lot of warnings concerning stereo chemistry will be printed. These warnings can be ignored.

```` ```
sdfGroovy.csh -in Novartis_GNF_NoModifier.sdf -out .sdf \
    -c 'v=tVal($mol,"PF proliferation inhibition 3D7 EC50 uM"); if(v.startsWith("<")) setVal($mol,"PF proliferation inhibition 3D7 EC50 uM",$v.replace("<",""))' \
| sdfSorter.csh -in .sdf -out .sdf \
    -numeric -sortTag "PF proliferation inhibition 3D7 EC50 uM" \
| sdfCluster.pl -in .sdf -out .sdf -radius 0.3 -nCpu 2 -- -AAPathSim DEFAULT \
| gzip -c >Novartis_GNF_NoModifier.AAPath.0.3.sdf.gz
``` ````

The output file will contain the following additional fields:

- clusterIdx   
  The index of the cluster to which the compound was assigned.
- centroidIdx   
  The index of the cluster of which this compound is the seed.
  Only present for seeds.
- NNSim   
  The similarity of this compound to its cluster seed.

## Producing data for figure S1

Note that due to the random selection this will not produce an
identical set of compounds and results.

```` ```
sdfGroovy.csh -in Novartis_GNF_NoModifier.sdf -out random.sdf -c 'Math.random()<0.025'
sdfMCSSNNFinder.csh -AAPathSim DEFAULT4 -in random.sdf -tabOutput vTab -out DEFAULTHung.tab -maxNeighbors 1000000
sdfMCSSNNFinder.csh -AAPathSim DEFAULT2 -in random.sdf -tabOutput vTab -out DEFAULT2.tab -maxNeighbors 1000000
``` ````
