# Supplementary material for: Atom-Atom-Path similarity and Sphere Exclusion clustering: tools for prioritizing fragment hits
Source: J Cheminform. 2015 Mar 25;7:11. doi: 10.1186/s13321-015-0056-8 (PMC4392110; doi:10.1186/s13321-015-0056-8)
Supplement: Additional file 3: — gCheminfoCommands_20150126.tgz. Zip file containing source code and compiled code for software described in this paper. Unpack the zip file into a directory on a UNIX server and follow the instructions in the = readme.md file to install the software. Step by step instructions on how to reproduce the clustering method on the Novartis-GNF Malaria Box dataset can be found in the examples/NovartisMalariaBox/=readme.md file. [file 13321_2015_56_MOESM3_ESM.zip › 13321_2015_56_MOESM3_ESM.tgz!/examples/NovartisMalariaBox/Novartis_GNF_Assay_Desc.doc]

Plasmodium falciparum strains 3d7 (drug-susceptible) and W2 (chloroquine-, quinine-, pyrimethamine-, cycloguanil-, and sulfadoxine-resistant), obtained from MR4, were tested in an erythrocyte-based infection assay for susceptibility to inhibition of proliferation by selected compounds (Malaria Box). Compounds were screened in a 12 point dose-response (1/2 log serial dilutions) in 1536-well format and concentrations ranged from either 12.5 µM to 0.0001 µM (DMSO solutions direct from powder stocks) or 1.25 µM to 0.00001 µM (compounds stored as DMSO stock solutions, standard for HTS). Parasite cultures (8 µL) at 0.3% parasitemia and 2.5% hematocrit were treated with compound for 72hr under low oxygen conditions (96% nitrogen, 3% carbon dioxide and 1% oxygen). After 72hr parasite growth is determined by measuring the nucleic acid content of the parasites with the fluorescent dye SybrGreen and plates were read on an Envision plate reader (Perkin Elmer). Known antimalarial drugs are used as reference compounds (mefloquine, pyrimethamine and artemisinin).

For both 3d7 and W2 P. falciparum assays the reconfirmation rate with compounds generated directly from powder stocks (EC_50_ within 5-fold of the HTS data) is ~85%. HTS hits are defined as EC_50_ < 2 µM on
3D7 or W2 strain and powder hits are defined as EC_50_ < 10 µM or 3D7 or W2 strain.

The effect of compound exposure in the Huh7 human hepatocellular carcinoma cell line is used as a measure of general compound cytotoxicity. Compounds were screened in a 12 point dose-response (1/2 log serial dilutions) in 1536 well format at concentrations ranging from 100 µM to 0.0003 µM (DMSO solutions direct from powder stocks) or 10 µM to 0.00003 µM (compounds stored as DMSO stock solutions). Cells are seeded at 500 cells per 5 µL media per well. Compounds were transferred the next day and cells are cultured for 72 hr to match the incubation length during the P. falciparum proliferation inhibition assay. Cellular viability is assessed using Cell Titer Glo (Promega).

The Huh7 assay uses a human hepatoma cell line as a measure of general compound cytotoxicity. Huh7 cells are seeded at 2500 cells per 50 µL media per well in a 384 well plate, compounds are transferred the next day and cells are cultured for 72 hr to match the incubation length during the Pf proliferation inhibition assay. Cellular toxicity is assessed using Cell Titer Glo.

IFI promiscuity index: The number is the ratio between the number of screens for which a compound is active and the total number screens in which it was tested. A compound is active if it has 50% or more inhibition in antagonist screens or 50% or more induction in agonist screens (compared to a agonist control) at the screening concentration, for most cases, it is 10 µM. >100 HTS campaigns were used in this analysis including biochemical and cell-based assays, though not all compounds were tested in all assays.
