# Supplementary material for: Atom-Atom-Path similarity and Sphere Exclusion clustering: tools for prioritizing fragment hits
Source: J Cheminform. 2015 Mar 25;7:11. doi: 10.1186/s13321-015-0056-8 (PMC4392110; doi:10.1186/s13321-015-0056-8)
Supplement: Additional file 3: — gCheminfoCommands_20150126.tgz. Zip file containing source code and compiled code for software described in this paper. Unpack the zip file into a directory on a UNIX server and follow the instructions in the = readme.md file to install the software. Step by step instructions on how to reproduce the clustering method on the Novartis-GNF Malaria Box dataset can be found in the examples/NovartisMalariaBox/=readme.md file. [file 13321_2015_56_MOESM3_ESM.zip › 13321_2015_56_MOESM3_ESM.tgz!/src/com/aestel/chemistry/openEye/fp/doc-files/hash.pdf]

Hash Function Efficiency

(This article is also available in Russian)

In article of Arash Partow "General Purpose Hash Function Algorithms" [http://www.partow.net/programming/hashfunctions/] several 32-bit algorithms are reviewed:

- rs — simple hash function from Robert Sedgwicks book 'Algorithms in C' [http://www.amazon.com/gp/product/0201514257/]
- js — bitwise hash function by Justin Sobel
- pjw — algorithm based on work by Peter J. Weinberger
- bkdr — hash function from Brian Kernighan and Dennis Ritchie’s book 'The C Programming Language' [http://www.amazon.com/gp/product/0131103628/]
- sdbm — algorithm of choice used in SDBM project
- djb — algorithm produced by Professor Daniel J. Bernstein
- dek — algorithm proposed by Donald E. Knuth in 'The Art Of Computer Programming' [http://www.amazon.com/gp/product/0201896850/]
- ap — algorithm produced by Arash Partow

Another five variants:

- faq6 — number 6 from FAQ by Bob Jenkins [http://burtleburtle.net/bob/hash/hashfaq.html]
- lookup3 — author Bob Jenkins [http://burtleburtle.net/bob/hash/]
- ly — proposed by Leonid Yuriev [http://leo.yuriev.ru/random] (congruential generator)
- rot13 — simple algorithm with circular shift, by Serge Vakulenko
- crc32 — standard checksum [http://www.w3.org/TR/PNG-CRCAppendix.html] with polynom $x^{32}+x^{26}+x^{23}+x^{22}+x^{16}+x^{12}+x^{11}+x^{10}+x^{8}+x^{7}+x^{5}+x^{4}+x^{2}+x+1$

Here are the C sources.

Test 1

To measure the efficiency of hash functions I prepared the following test data:

- American dictionary [/lib/exe/fetch.php/proj/hash/usdict.gz?id=proj%3Ahash%3Aefficiency-en&cache=cache] from Ispell project, 62075 words.
- Russian dictionary [/lib/exe/fetch.php/proj/hash/rudict.gz?id=proj%3Ahash%3Aefficiency-en&cache=cache] from Ispell project, 128900 words.
- A list of symbols [/lib/exe/fetch.php/proj/hash/symbols.gz?id=proj%3Ahash%3Aefficiency-en&cache=cache], extracted from all libs on my linux workstation (libc.a and others), 136073 words.

Total volume after merging is **326797** different words.

For every word a 32-bit hash value was computed, and counted a number of collisions.

Algorithm	Collisions
**rs**	9
**js**	98
**pjw**	1315
**bkdr**	11
**sdbm**	14
**djb**	83
**dek**	308
**ap**	16
**ly**	9

**rot13**	7
**faq6**	14
**lookup3**	9
**crc32**	13

A list of collisions for rs, bkdr, sdbm, ap, ly, rot13, faq6, lookup3 and crc32 is available here.

Algorithms with minimal collisions:

- rot13 — one circular shift (rotation) and addition
- lookup3 — one shift and addition (or more)
- ly — one multiplication and addition
- rs — two multiplications

Results are presented on picture. Two outsiders — pjw and dek — exceed the limits of Y axis.

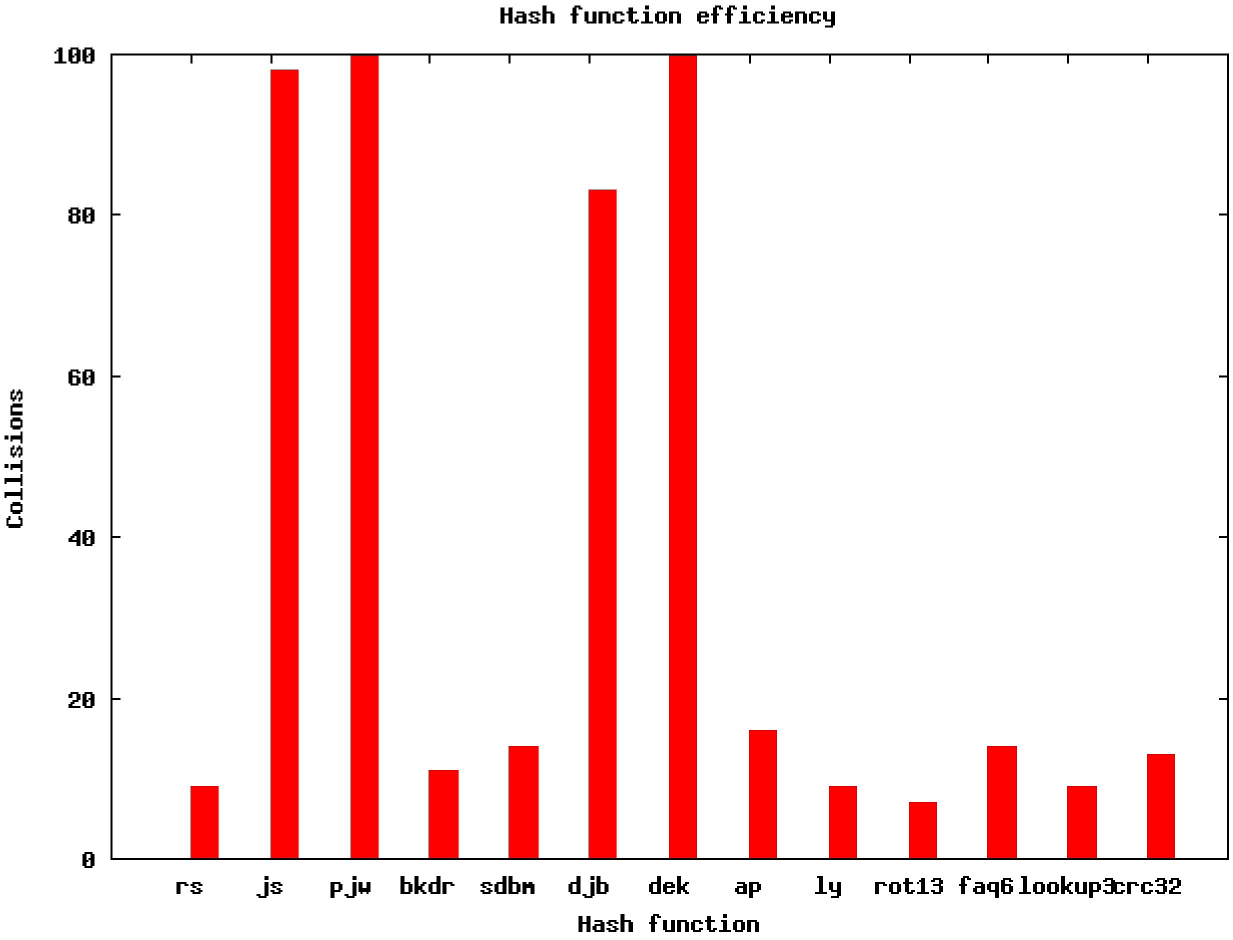

Test 2

In previous test, all data had MSB unchanged. For the second test another data set was selected:

- German dictionary [/lib/exe/fetch.php/proj/hash/dedict.gz?id=proj%3Ahash%3Aefficiency-en&cache=cache] from Ispell project, 39612 words.
- Hungarian dictionary [/lib/exe/fetch.php/proj/hash/hudict.gz?id=proj%3Ahash%3Aefficiency-en&cache=cache] from Ispell project, 211880 words.
- Italian dictionary [/lib/exe/fetch.php/proj/hash/itdict.gz?id=proj%3Ahash%3Aefficiency-

en&cache=cache] from Ispell project, 37268 words.
- Swedish dictionary [/lib/exe/fetch.php/proj/hash/sedict.gz?id=proj%3Ahash%3Aefficiency-en&cache=cache] from Ispell project, 24019 words.

Total volume after merging is **310595** different words.

Algorithms js, pjw, djb и dek were excluded from testing.

Algorithm	Collisions
rs	5
bkdr	14
sdbm	14
ap	32
ly	8
rot13	10
faq6	13
lookup3	14
crc32	15

Algorithms with minimal collisions:

- rs — two multiplications
- ly — one multiplication and addition
- rot13 — one circular shift (rotation) and addition

Results are presented on picture.

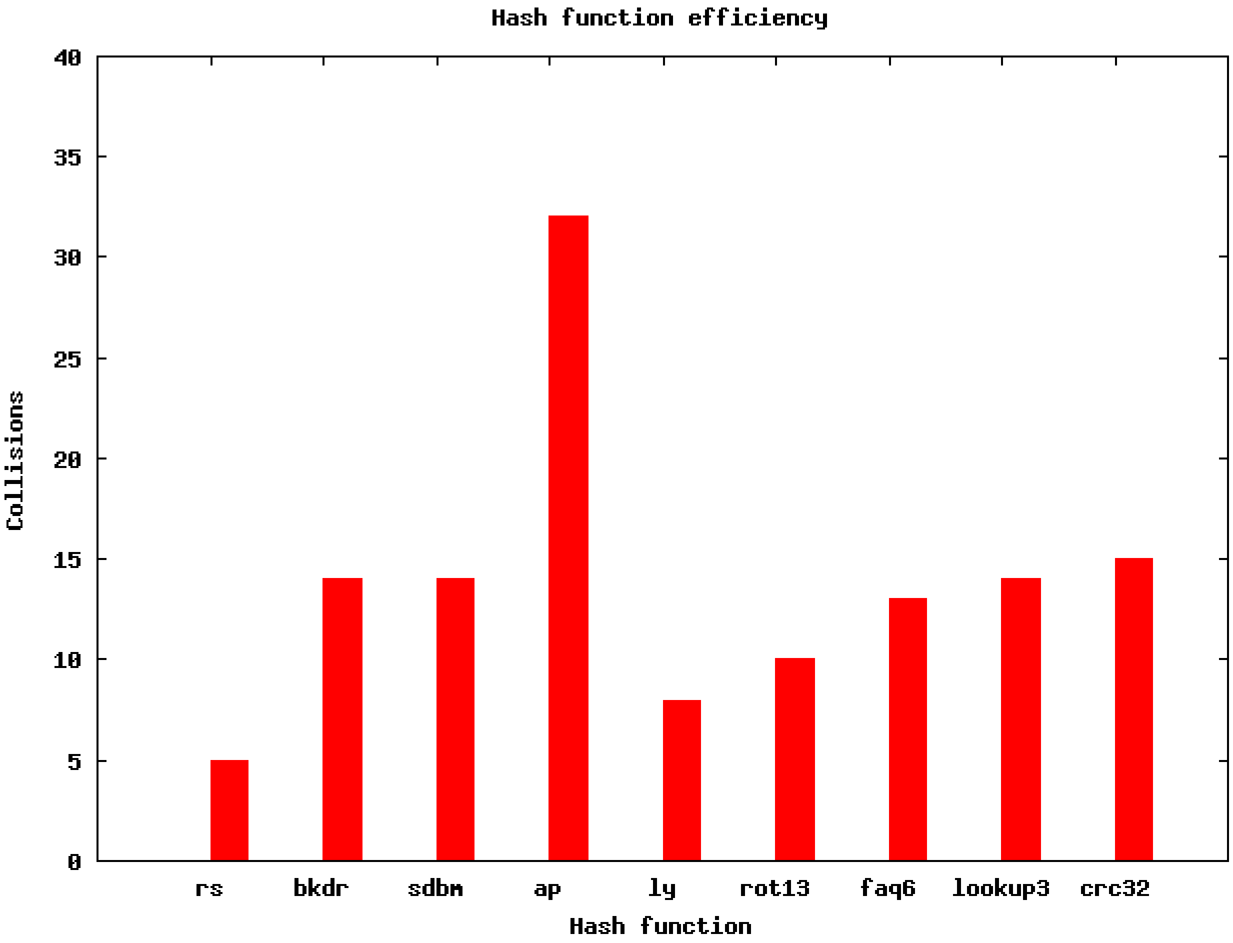

proj/hash/efficiency-en.txt · Последние изменения: 2006/06/18 01:45 vak
