# Supplementary material for: Atom-Atom-Path similarity and Sphere Exclusion clustering: tools for prioritizing fragment hits
Source: J Cheminform. 2015 Mar 25;7:11. doi: 10.1186/s13321-015-0056-8 (PMC4392110; doi:10.1186/s13321-015-0056-8)
Supplement: Additional file 3: — gCheminfoCommands_20150126.tgz. Zip file containing source code and compiled code for software described in this paper. Unpack the zip file into a directory on a UNIX server and follow the instructions in the = readme.md file to install the software. Step by step instructions on how to reproduce the clustering method on the Novartis-GNF Malaria Box dataset can be found in the examples/NovartisMalariaBox/=readme.md file. [file 13321_2015_56_MOESM3_ESM.zip › 13321_2015_56_MOESM3_ESM.tgz!/src/com/aestel/chemistry/openEye/fp/doc-files/hash2.pdf]

S M A L L C O D E
53 4D 41 4C 4C 43 4F 44 45
Code optimization, assembly language and C programming for Win32

- Home

- **Popular Articles**
  - Hash functions benchmark
  - Self-extracting executables
  - What your compiler can do 4u
  - Win32 assembly cheat sheet
  - x86 machine code statistics

Search

- Recent Comments
  - Adam Messinger: In your equal any example, will the first bit really be 1? The Y in "You Drive Me Mad" is...
  - ace: I haven't read the official book but it appears that it can contain what I was talking about:...
  - Rolland: This is plain wrong. The string passed in argument is const char, so should not be modified.If the string...
  - nairam: Hello. All is A4 format: x86 registers: www.nairam.sk/pc01.pdf x86 instructions: www.nairam.sk/pc03.pdf...
  - ace: There was once somewhere on the web some shorter version of antipatterns text which is probably not present in...
- Categories
  - All
  - Off-topic
  - Optimization tricks
  - Machine code
  - News and links
  - Win32 programming
  - Assembly language
  - Algorithms
  - Code organization
- Archives
  - July 2008
  - June 2008
  - April 2008
  - March 2008
  - February 2008
  - January 2008
  - December 2007
  - November 2007
  - July 2007
  - June 2007
  - April 2007
  - March 2007
  - February 2007

- January 2007
- December 2006
- November 2006
- October 2006
- September 2006
- August 2006
- July 2006
- June 2006
- May 2006
- April 2006
- March 2006

- Subscribe to this blog
  - RSS
  - Comments RSS

January 2008

M	T	W	T	F	S	S
	1	2	3	4	5	6
7	8	9	10	11	12	13
14	15	16	17	18	19	20
21	22	23	24	25	26	27
28	29	30	31			

« Dec Feb »

-
- Pages
  - About author

January 22, 2008

Hash functions: An empirical comparison

Filed under: Algorithms — Peter Kankowski @ 11:12 am

There are two classes of hash functions:

- multiplicative hash functions, which are simple and fast, but have a high number of collisions;
- more complex functions, which have better quality, but take more time to calculate.

Hash function benchmarks usually include theoretical metrics such as the number of collisions or distribution uniformity (see, for example, hash function comparison in the Red Dragon book). Obviously, you can have better distribution with more complex functions, so they are winners in these benchmarks.

The question is **whether using complex functions gives you a faster program**. Is the price of collisions high enough to justify the usage of more complex function? In this article, I will try to answer this question.

How does a multiplicative hash function work?

Any multiplicative hash function is a special case of the following algorithm:

```
UINT HashMultiplicative(const CHAR *key, SIZE_T len) {
```

```
   UINT hash = INITIAL_VALUE;
   for(UINT i = 0; i < len; ++i)
      hash = M * hash + key[i];
   return hash % TABLE_SIZE;
}
```

(Sometimes XOR operation is used instead of addition, but it's not really important.) The hash functions differ only by values of INITIAL_VALUE and multiplier (M). For example, the popular **Bernstein's function** uses INITIAL_VALUE of 5381 and M of 33; **Kernighan and Ritchie's function** uses INITIAL_VALUE of 0 and M of 31.

A multiplicative function works by adding together the letters weighted by powers of multiplier. For example, the hash for the word *TONE* will be:

```
INITIAL_VALUE * M^4  +  'T' * M^3  +  'O' * M^2  +  'N' * M  +  'E'
```

Let's enter several similar strings and watch the output of the functions:

```
     Bernstein Kernighan
        (M=33)     (M=31)
 too   b88af17     1c154
 top   b88af18     1c155
 tor   b88af1a     1c157
 tpp   b88af39     1c174
a000  7c9312d6    2cd22f
a001  7c9312d7    2cd230
a002  7c9312d8    2cd231
a003  7c9312d9    2cd232
a004  7c9312da    2cd233
a005  7c9312db    2cd234
a006  7c9312dc    2cd235
a007  7c9312dd    2cd236
a008  7c9312de    2cd237
a009  7c9312df    2cd238
a010  7c9312f7    2cd24e
   a     2b606        61
  aa    597727       c20
 aaa   b885c68     17841
```

Too and *top* are different in the last letter only. The letter P is the next one after O, so the values of hash function are different by 1 (1c15**4** and 1c15**5**, b88af1**7** and b88af1**8**). Ditto for *a000..a009*.

Now let's compare *top* with *tpp*. Their hashes will be:

```
INITIAL_VALUE * M^3 + 'T' * M^2 + 'O' * M + 'P'
INITIAL_VALUE * M^3 + 'T' * M^2 + 'P' * M + 'P'
```

The hashes will be different by `M * ('P' - 'O') = M`. Similarly, when the first letters are different by X, their hashes will be different by `X * M^2`.

When there are less than 33 possible letters, Bernstein's function will pack them into a number (similar to [Radix40 packing scheme]). For example, hash table of size 33^3 will provide perfect hashing (without any collisions) for all three-letter English words written in small letters. In practice, the words are longer and hash tables are smaller, so there will be some collisions (situations when different strings have the same hash value).

If the string is too long to fit into the 32-bit number, the first letters will still affect the value of the hash function, because the multiplication is done modulo 2^32 (in a 32-bit register). The multiplier is chosen to have no common divisors with 2^32 (in other words, it must be odd), so the bits will not be just shifted away.

For table size less than 2^16, we can improve the quality of hash function by XORing high and low words, so that more letters will be taken into account:

```
   return hash ^ (hash >> 16);
```

There are no exact rules for choosing the multiplier, only some heuristics:

- the multiplier should be large enough to accommodate most of the possible letters (e.g., 3 or 5 is too small);
- the multiplier should be fast to calculate with shifts and additions [e.g., 33 * hash can be calculated as (hash << 5) + hash];
- the multiplier should be odd for the reason explained above;
- prime numbers are good multipliers.

My own function

Just like many people who wrote about hash functions, I could not resist the temptation of inventing my own hash :). I tried a smaller multiplier (17), which allows packing more letters in the hash value:

```
// My hash function
UINT Hash17(const CHAR *key, SIZE_T len) {
    UINT hash = 0;
    for(UINT i = 0; i < len; ++i) {
        hash = 17 * hash + (key[i] - ' ');
    }
    return hash ^ (hash >> 16);
}
```

Another change was subtracting a space from each letter to cut off the control characters in the range 0x00..0x1F. If the hash keys are long and contain only Latin letters and numbers, the letters will be less frequently shifted out, and the overall number of collisions will be lower. You can even subtract 'A' when you know that the keys will be only English words.

Complex hash functions

These functions do a good job of mixing together the bits of the source word. The change in one input bit changes a half of the bits in the output (that's called avalanche effect), so the result looks completely random:

```
     Paul Hsieh One At Time
 too   3ad11d33  3a9fad1e
 top   78b5a877  4c5dd09a
 tor   c09e2021  f2aa9d35
 tpp   3058996d  d5e9e480
a000   7552599f  ed3859d8
a001   3cc1d896  fef7fd57
a002   c6ff5c9b  08a610b3
a003   dcab7b0c  1a88b478
a004   780c7202  3621ebaa
a005   7eb63e3a  47db8f1d
a006   6b0a7a17  b901717b
a007   cb5cb1ab  caec1550
a008   5c2a15c0  e58d4a92
a009   33339829  f75aee2d
a010   eb1f336e  bd097a6b
   a   115ea782  ca2e9442
  aa   008ad357  7081738e
 aaa   7dfdc310  ae4f22ec
```

To achieve this behavior, the hash functions include a lot of shifts, XORs, and additions. But are these tricks

really needed? What is faster: tolerating the collisions and resolving them with linear probing, or avoiding them with a more complex function?

Test conditions

For the benchmark, I used the simplest linear probing algorithm. The items filled 1/4…1/2 of the table (e.g., for 500 words, the hash table had 1024 items).

Memory allocation and other "heavy" functions were excluded from the benchmarked code. The RDTSC instruction was used for benchmarking. The test was conducted on Pentium-M 1.5 GHz, 1.25 GB of RAM under Windows XP SP2.

The benchmark inserts some keys in the table, then looks them up in the same order as they were inserted. The test data include:

- the list of common words from Wiktionary (500 items);
- the list of Win32 functions from Colorer syntax highlight scheme (1992 items);
- 500 names from *a000* to *a499* (imitates the names in auto-generated source code);
- the list of common words with a long prefix and postfix (*my_hash_function_test*).

Results

	Common words	Win32 functions	Numbers	Prefix	Postfix
Bernstein	146.3 261	888.4 889	427.2 8030	325.5 214	316.5 226
Kernighan & Ritchie	141.6 194	896.6 1006	842.2 19533	338.0 274	323.3 256
x17 (my own)	137.1 193	848.9 1002	81.4 340	313.1 244	299.8 228
x65599	140.0 250	836.5 816	206.3 3158	317.9 200	314.4 316
FNV-1a	151.3 262	952.3 1021	87.2 207	367.5 233	355.3 237
Weinberger	169.4 291	1203.1 908	274.3 4360	482.8 304	471.4 309
Paul Hsieh	158.1 268	843.4 921	112.5 342	289.7 279	275.5 281
One At Time	162.6 239	1036.2 1003	103.0 267	394.9 246	382.9 265
Arash Partow	165.8 248	1043.8 931	1040.3 20860	415.8 279	393.2 199

Large figures denote execution time in thousands of clock cycles (lower number is better), and small figures denote the number of collisions. The 3 best results in each test are highlighted with green color.

The function by Kernighan and Ritchie is from their famous book "The C programming Language", 3rd edition; Weinberger's hash and a hash with multiplier 65599 are from the Red Dragon book. Bernstein's function is said to be published by him on comp.lang.c, but I was unable to find his original post.

As you can see from the table, the function with the lowest number of collisions is not always the fastest one. For example, compare FNV-1a and x17 in the "numbers" test.

The winners are the function by Paul Hsieh, the function with multiplier 65599, and my own function.

Paul Hsieh's function is tuned for long keys (he originally benchmarked it for the keys of 256 bytes). That's why it is the winner for the postfix and prefix tests. Note that it does not provides the best number of collisions for these tests, but it does have the best time, which means that the function is faster than the others.

Hsieh's function is suboptimal for short keys ("common words" and "numbers"). You may want to use a

simpler function for a word counting program, a compiler, or another application that typically handles short keys. Anyway, the function by Paul Hsieh provides good, conservative performance for all kind of keys.

The function with multiplier 65599 shows good results in all tests except “numbers”. It’s said to be used in gawk, sdbm, and other Linux programs.

My own function is fast in all tests, but it needs additional study on a broader dataset to confirm that it will behave nicely in all circumstances.

As you can see, **more complex hash functions not always provide better performance**. The speed of Weinberger’s function and *One At Time* did not impressed me at all, and the function by Paul Hsieh was not always better than a simple multiplicative hash.

Download the test program (.zip archive, 53 KB)

• • •

Hash functions Series

1. Hash functions: An empirical comparison
2. Hash functions: additional tests
3. Hash functions, part 3
4. Murmur hash

9 Comments »

1. Hi.

   Have you tried to compare the hash-functions against CRC32? That would be interesting!

   Some DSP's can already do galois multiplies which is the slow part of CRC. For PC we'll have cheap CRC in the future when the new SSE becomes mainstream (hopefully).

   Cool blob btw..

   Comment by nils — January 28, 2008 @ 8:32 pm

2. CRC implementation on x86 is slower than the other hash functions (see Paul Hsieh's tests). With SSE4, it should be faster, and it would be interesting to compare them in future. Let's wait for SSE4 :).

   Comment by Peter Kankowski — January 29, 2008 @ 8:35 am

3. As far as I know, the authors of the traditional hash functions that you presented made them under the assumption that the size of the table is a **prime**, not some "round" number like 1024. They counted on the modulo step to spread the resulting values. So when you use 1024 as the table size, of course their functions don't fare good. Can you try the table sizes that they would use and share these results with us?

   Comment by ace — January 31, 2008 @ 12:29 am

4. Thank you for the idea, I will test it this weekend (and also CRC function that Nils proposed). However, modulus of prime number will be much slower than (hash % 1024), which is optimized to

(hash & 1023), so I'm not sure which one will be faster.

Comment by Peter Kankowski — January 31, 2008 @ 8:36 am

5. Hi Peter nice article wanted to mention a few things though:

   Most of the function presented there produce entropy of some level at a quantity of 32-bits. What you are doing by mod'ing by 1024 is ignoring the 22-bit higher bits. You should integrate them back into the result somehow.

   A true test would not quantize the values. Instead it would just make a list of already generated values and see if those generated values occur again within the period. The period being the size of the "common words" etc.

   Another good test is to see the avalanching abilities of the functions. In this case you only change 1 bit in the input and then see how many of the output bits change. The average should be close to about half the number of output bits(eg: 16 bits in the case of 32-bit outputs)

   Another good thing to remember is to use random values, than only English or some such as you will see that in the case of English only certain bits in a byte are likely to change, how many times do we use control characters in English words?

   I believe if you follow the above you may see different results...

   Comment by Arash Partow — February 3, 2008 @ 5:38 am

6. Arash, I think that Peter didn't want to investigate "avalanching abilities" or something like that -- his goal was to evaluate the functions on the "good enough" principle in some specific example.

   Comment by acd — February 3, 2008 @ 8:15 pm

7. The next part of this article

   Pingback by smallcode » Blog Archive » Hash functions: additional tests — February 4, 2008 @ 7:42 pm

8. Arash, for multiplicative functions, the tests mix together higher and lower 16 bits with XOR, so higher bits are not ignored.

   My purpose was to benchmark the hash functions in close-to-real-life scenario. Theoretical tests will give a completely different result.

   For example, cryptographic hashes such as MD5 will give a perfect distribution, but they are impractically slow for hash tables. Good results in a theoretical test not always mean a fast hash function.

   Comment by Peter Kankowski — February 4, 2008 @ 7:53 pm

9. Have you reviewed “Performance in Practise of String Hashing Functions” (Jobel & Ramakrishna, 1997) at all? They have some interesting data on performance of various classes of hash functions. I've used their conclusions myself with good results, see here: http://stochasticgeometry.wordpress.com/2008/03/29/cache-concious-hash-tables/

   Comment by Mark Dennehy — April 2, 2008 @ 8:29 am

Comments RSS • TrackBack URI

Leave a comment

Name (required)

Mail (will not be published) (required)

Website

You can use <b>, <i>, and <pre> tags in your comment. If you insert some C code, please put [ccode] and [/ccode] tags around it.

Submit Comment

Hosting is generously provided by: Weblogs.us • Theme by: Wench
